# DNA methylation changes during preimplantation development reveal inter-species differences and reprogramming events at imprinted genes

**DOI:** 10.1186/s13148-020-00857-x

**Published:** 2020-05-11

**Authors:** Elena Ivanova, Sebastian Canovas, Soledad Garcia-Martínez, Raquel Romar, Jordana S. Lopes, Dimitrios Rizos, Maria J. Sanchez-Calabuig, Felix Krueger, Simon Andrews, Fernando Perez-Sanz, Gavin Kelsey, Pilar Coy

**Affiliations:** 1 Epigenetics Programme, The Babraham Institute, Cambridge, CB22 3AT UK; 2 Physiology of Reproduction Group, Departamento de Fisiología, Universidad de Murcia, Campus Mare Nostrum, 30100 Murcia, Spain; 3 Instituto Murciano de Investigación Biosanitaria, IMIB-Arrixaca-UMU, 30120 Murcia, Spain; 4 Departamento Reproducción Animal, INIA, Madrid, Spain; 5 Bioinformatics Group, The Babraham Institute, Cambridge, CB22 3AT UK; 6 Centre for Trophoblast Research, University of Cambridge, Cambridge, CB2 3EG UK

**Keywords:** DNA methylation, Embryo, Epigenetic, Imprinting

## Abstract

Preimplantation embryos experience profound resetting of epigenetic information inherited from the gametes. Genome-wide analysis at single-base resolution has shown similarities but also species differences between human and mouse preimplantation embryos in DNA methylation patterns and reprogramming. Here, we have extended such analysis to two key livestock species, the pig and the cow. We generated genome-wide DNA methylation and whole-transcriptome datasets from gametes to blastocysts in both species. In oocytes from both species, a distinctive bimodal methylation landscape is present, with hypermethylated domains prevalent over hypomethylated domains, similar to human, while in the mouse the proportions are reversed.

An oocyte-like pattern of methylation persists in the cleavage stages, albeit with some reduction in methylation level, persisting to blastocysts in cow, while pig blastocysts have a highly hypomethylated landscape. In the pig, there was evidence of transient de novo methylation at the 8–16 cell stages of domains unmethylated in oocytes, revealing a complex dynamic of methylation reprogramming. The methylation datasets were used to identify germline differentially methylated regions (gDMRs) of known imprinted genes and for the basis of detection of novel imprinted loci. Strikingly in the pig, we detected a consistent reduction in gDMR methylation at the 8–16 cell stages, followed by recovery to the blastocyst stage, suggesting an active period of imprint stabilization in preimplantation embryos. Transcriptome analysis revealed absence of expression in oocytes of both species of ZFP57, a key factor in the mouse for gDMR methylation maintenance, but presence of the alternative imprint regulator ZNF445. In conclusion, our study reveals species differences in DNA methylation reprogramming and suggests that porcine or bovine models may be closer to human in key aspects than in the mouse model.

## Significance statement

Our study provides extensive genome-wide datasets that detail at single-base resolution the profound resetting of epigenetic information experienced by gametes and preimplantation embryos. It also provides new insights into establishment and maintenance of DNA methylation imprints. Data are of utmost importance as a reference for species similarity and differences in epigenetic mechanisms occurring during a critical window of development. We consider our study to be of interest to scientists working in developmental biology, evolution, genetics and epigenetics, and also for physicians, patients using ART and researchers seeking to understand how the environment during preimplantation development may have consequences on the epigenome, with further effects after birth and during adulthood.

## Introduction

Genes are inherited from parents and, once their specific sequence after recombination during meiosis is established, they remain almost unaltered through cell divisions during an individual’s life, unless external agents or replication mistakes act on them producing mutations. However, changes in epigenetic information, associated with states of gene activity, are at least one order of magnitude more frequent than genetic changes [3] and more susceptible to environmental conditions. Epigenetic states are particularly dynamic during gametogenesis and early embryonic development when extensive reprogramming takes place [7]. Thus, their correct establishment during preimplantation development may be crucial to avoid immediate or future alterations in the offspring’s health. According to the DOHAD hypothesis [1], the knowledge of the epigenetic marks that are rewritten during early embryonic development of an individual may serve to predict predisposition to certain diseases in adult life. Therefore, it is important to establish the normal patterns, as well as the expected changes during the life-course, of these marks in each species, and find out the similarities and differences between them.

The first genome-scale DNA methylation maps of early embryo development were described over 7 years ago in the mouse [32, 63, 66], providing a nucleotide-resolution view of the unique regulatory wave of global demethylation that had previously been observed by immunofluorescence [59]. Similar principles were subsequently found in human embryos [22, 65]. These and other studies reveal, for example, the distinctive DNA methylation landscape of the mouse and human oocyte, comprising alternating hyper- and hypomethylated domains and predominant gene-body methylation [32, 44, 72]. However, a recent single-cell study has suggested that methylation reprogramming in the cleavage embryo could be more dynamic than previously thought, with remethylation and demethylation events coexisting and affecting different genomic regions during preimplantation development [77].

It has been generally assumed that imprinted genes, which are differentially methylated in the sperm and oocyte, represent an exception to the genome-wide reprogramming events and maintain their methylation marks during preimplantation stages. This persistence of monoallelic methylation is key to ensuring robust monoallelic expression of these genes and the normal development of the embryo [26, 34, 53, 66]. However, it has not been fully addressed whether this methylation maintenance is a consequence of a complete lack of reprogramming of these genes or whether they are also affected by demethylation and remethylation waves but are rapidly subjected to imprinting stabilization. Similarly, the identification of germline differentially methylated regions (gDMRs) in different species that maintain their methylation levels through these first cleavage divisions would be crucial to understand the molecular basis of preimplantation development and, consequently, to improve the efficiency and safety of the reproductive processes, especially when assisted reproductive technologies (ART) are used.

In order to describe the methylation landscape of preimplantation embryonic development with a multispecies approach, and determine common and divergent patterns, genome-scale, single-base resolution DNA methylation data were obtained from oocytes, spermatozoa, 2–4 cell embryos, 8–16 cell embryos, morulae and blastocysts of two major livestock species (porcine *Sus scrofa* and bovine *Bos taurus*). Datasets from previously published studies on human and mouse were used for comparison. In addition, transcriptome analysis by RNA-seq was performed on porcine and bovine samples collected at the same timepoints to assess correlations with promoter methylation.

## Results

### Global DNA methylation dynamics in early porcine and bovine embryos and comparison with mouse and human

In order to characterise the genome-wide patterns of DNA methylation during pre-implantation development in two major livestock species—cow and pig—we generated post-bisulfite adaptor tagging (PBAT) DNA libraries from spermatozoa, oocytes, 2-4 cell cleavage stage embryos, 8-16 cell cleavage stage embryos, morulae and blastocysts (day 6.5–7) from both species. The sequencing output of all libraries (including inferred bisulfite conversion rates) is given in Supplementary Table 1a,b. Replicates within groups were clustered together on the PCA plot (Supplementary Figure 1). After merging the replicates by stage, CpG coverage rates ranged from 61 to 87% of CpGs with > 1 read, and 4–30% of CpGs with > 5 reads in the PBAT libraries (Supplementary Figure 2) in pig; for the merged scPBAT dataset from bovine oocytes, 61% of genomic CpGs were represented, and CpG coverage from other stages ranged from 62 to 85% of CpG with > 1 read, and 2–42% of CpGs with > 5 reads (Supplementary Figure 2). Most downstream analysis was performed over features such as 100-CpG windows, and not at individual CpGs, to enable us to aggregate sufficient methylation calls to accurately quantify methylation across these features.

**Fig. 1:**
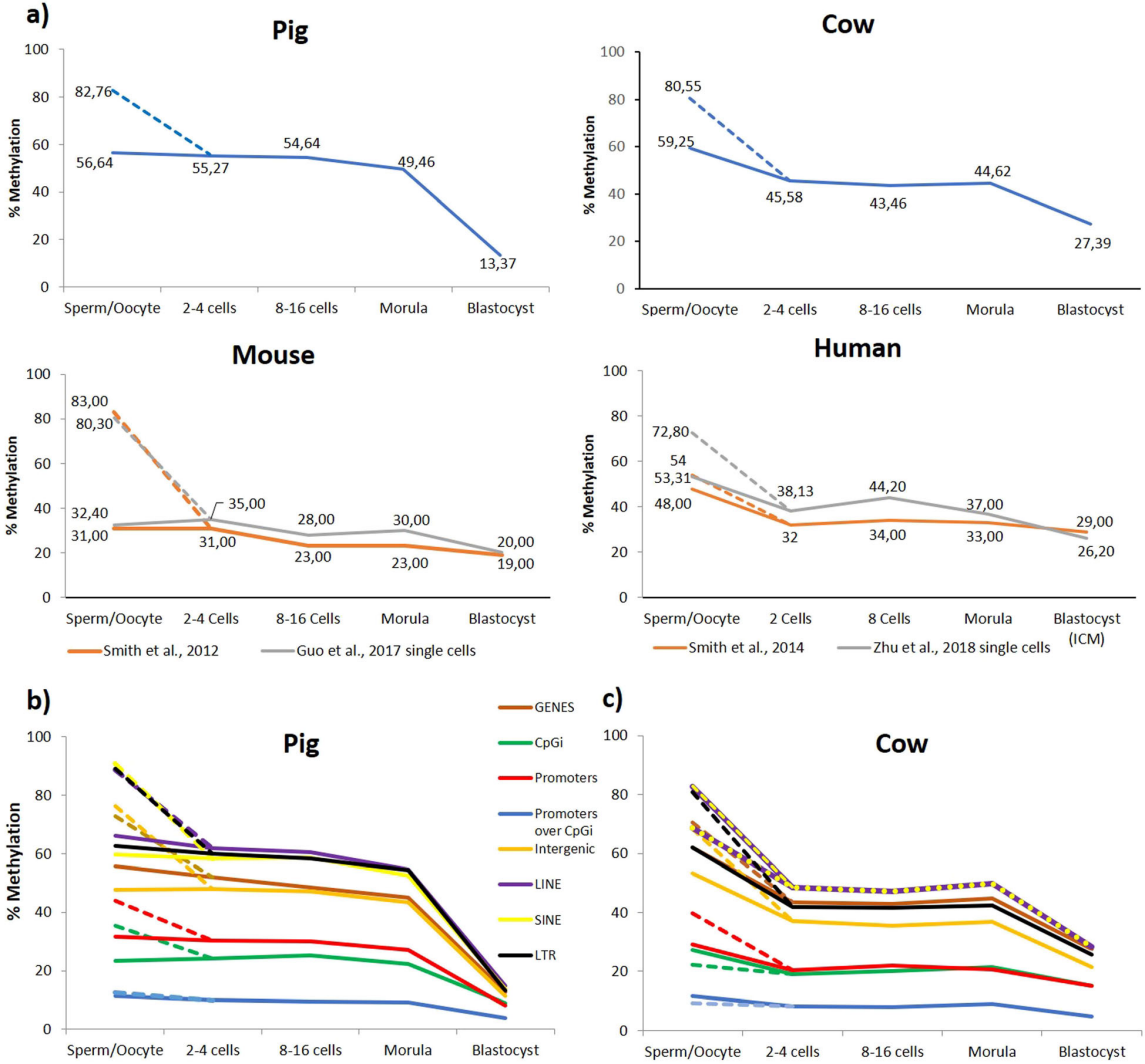
Changes in total genomic CpG methylation from gametes to blastocyst. **a** Line charts show global DNA methylation across the indicated stages in pig, cow, mouse and human. The mouse datasets are from Smith et al. [66] and Guo et al. [21]; the human datasets from Smith et al. [65] and Zhu et al. [77] Global DNA methylation at specific genomic features is shown for porcine (**b**) and bovine (**c**) samples. For the transitions from gametes to the 2–4 cell stages, dotted lines represent sperm data, and solid lines correspond to oocyte data

**Fig. 2:**
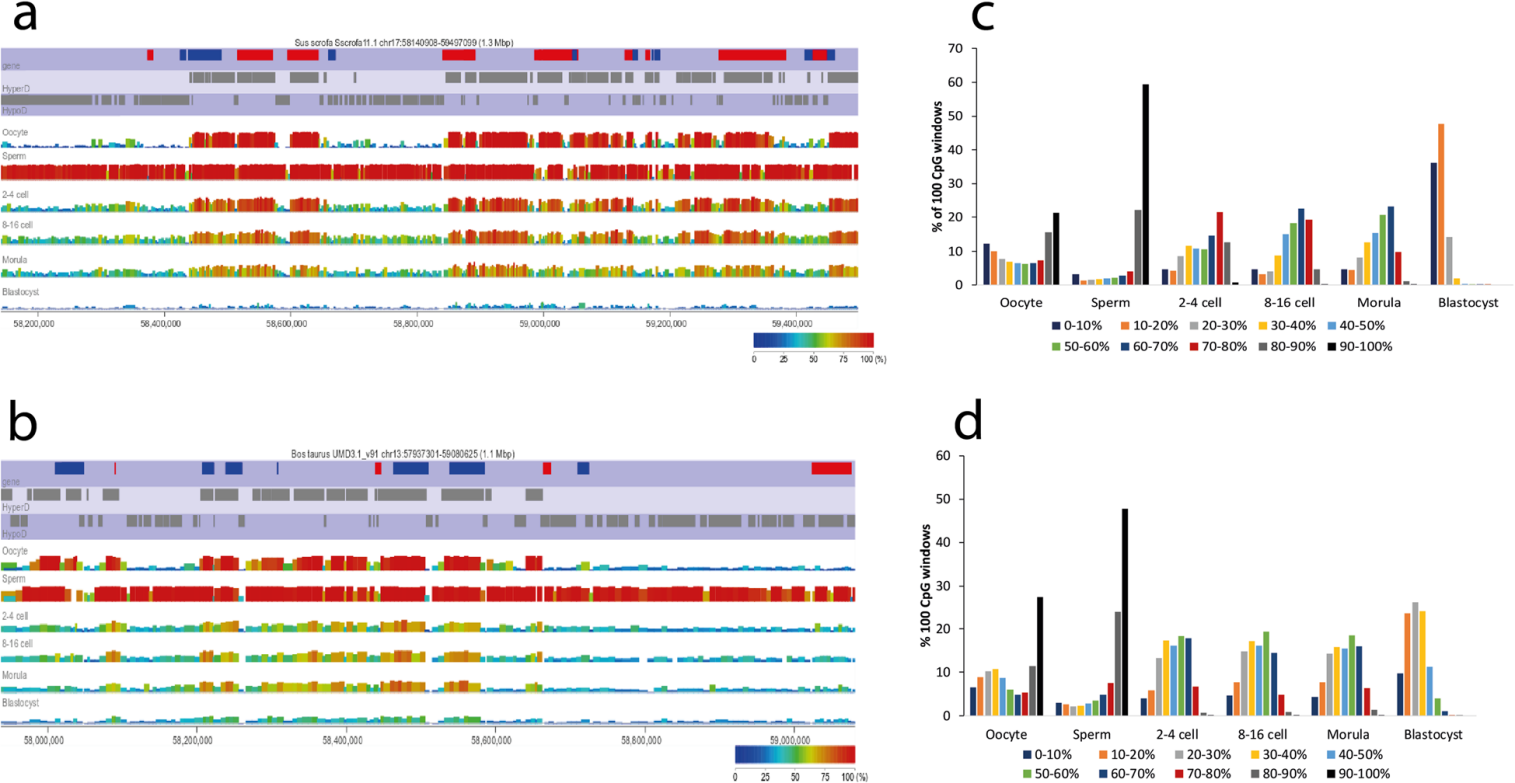
Changes in the CpG methylation landscape from gametes to blastocyst in pig and cow. Screenshot of Seqmonk genome browser at regions of conserved synteny in porcine chromosome 17 (**a**) and bovine chromosome 13 (**b**) centred on the GNAS locus. For the profiles for each stage, each vertical bar represents the methylation value of a single, non-overlapping 100-CpG tile, with methylation indicated by the height of the bar and the colour-coding. At the top, the track ‘gene’ indicates the location of genes, with those marked red being transcribed from left to right, and those marked blue from right to left; HyperDomains (HyperD) and HypoDomains (HypoD) are indicated by bar bars. Histograms of the percentage of 100-CpG windows binned according to the given methylation levels in porcine (**c**) and bovine (**d**) samples. The data in **c** are based on 253122 informative 100-CpG windows and in **d** on 256422 100-CpG windows

Global methylation levels exhibited similar trajectories from the gametes to the blastocyst in the two species, and these patterns were grossly similar to what has been described in mouse and human preimplantation embryos [22, 32, 44, 65], but quantitative differences were readily apparent (Fig. 1a). In all species, sperm are highly methylated, and oocytes have an intermediate methylation level. The reduced global methylation at the 2–4 cell stages compared with sperm is consistent with substantial demethylation after fertilisation. Across the cleavage stages, methylation is generally stable but with different absolute levels in the four species; e.g., 54.6% methylation at the 8–16 cell stages in the pig contrasting with a much lower level in the mouse at this stage (28.0%). In human, a peak in methylation level at the 8-cell stage has been taken as evidence for ongoing de novo methylation [77]. This is not seen in global methylation either in cow or pig, but below we show evidence for de novo methylation in pig. In all species, methylation is lowest at the blastocyst stage, with the pig showing the most precipitate decline between morula and blastocyst stages (49.5% to 13.4%). When segregating the genome into specific annotations, it is clear that all genomic features follow similar trends, although their averaged methylation levels vary markedly. In both species, promoter CpG islands consistently have the lowest methylation, while interspersed repeats (SINEs and LINEs) have the highest (Fig. 1b, c). The general transitions in methylation are also apparent in genome browser views, which reveal the contrast between highly methylated sperm and the mosaic methylation pattern of oocytes (alternating hyper- and hypomethylated domains) and the persistence of an oocyte-like pattern of methylation in the cleavage stages, albeit with some reduction in methylation level. This oocyte-like pattern appears to persist weakly even in cow blastocysts, whereas the genome in pig blastocysts has very low methylation with little apparent structure (Fig. 2a, b; Supplementary Figure 3).

For a more detailed evaluation of methylation dynamics, we defined non-overlapping tiles of 100 CpGs over both genomes, yielding 287679 and 268407 probes in the pig and cow genomes, respectively. Our level of sequence coverage allowed us to quantify methylation levels of over 90% of these tiles at all stages. Also, inspection of methylation levels of 100-CpG tiles revealed qualitative differences between the two species. In the pig, oocytes have a bimodal distribution of methylation, with the greatest numbers of 100-CpGs tiles in the unmethylated (0–10%) or fully-methylated (80–90%, 90–100%) fractions, whereas bovine oocytes have a far higher proportion of fully methylated tiles and proportionately more partially methylated (the 10–20%, 20–30%, 30–40% and 40–50% bins; Fig. 2c, d). Conversely, and consistent with the global methylation level, over the cleavage stages pig embryos have a higher proportion of highly methylated tiles (60–70, 70–80% range) than cow, but strikingly in the pig blastocyst there is a very small fraction of tiles with > 20% methylation.

### Conserved pattern of gene body methylation in pig and cow oocytes

**Fig. 3:**
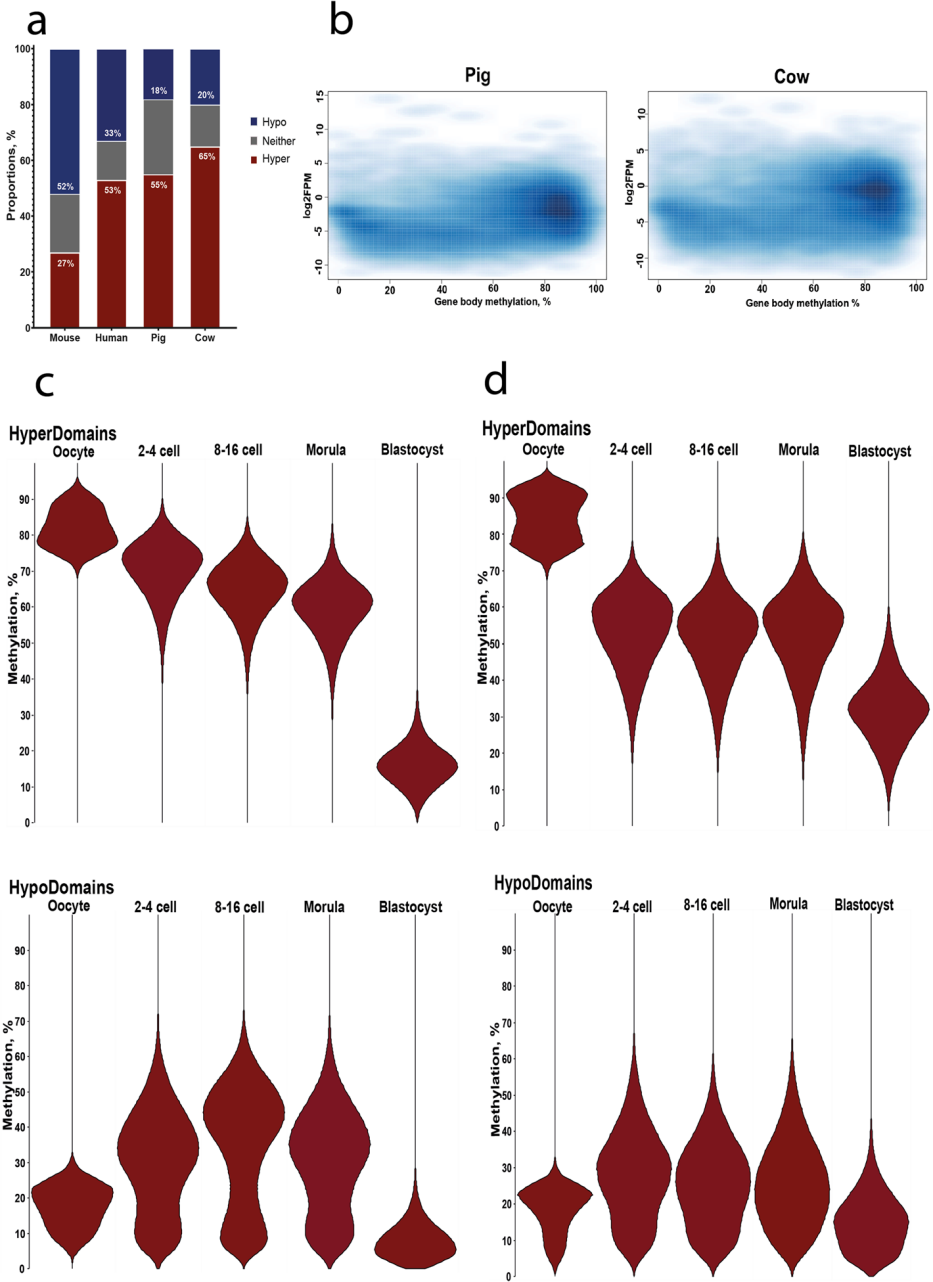
Properties of hyper- and hypomethylated domains in pig and cow. **a** Stacked bar chart of the percentage genome coverage of hypermethylated domains (HyperDomains; ≥ 75% methylation) and hypomethylated (HypoDomains; ≤ 25%) in oocytes from pig, cow, mouse and human. **b** Correlation between gene body methylation and gene expression in pig and cow oocytes. **c** Violin plots showing distribution of DNA methylation values (%) of oocyte HyperDomains and HypoDomains across the indicated stages in pig (**c**) and cow (**d**). The data in **c** are based on 55380 HyperDomains and 54959 HypoDomains and in **d** on 40453 HyperDomains and 57969 HypoDomains

Because of the apparent persistence of an oocyte-like pattern of methylation in embryos in both species (Fig. 2a, b), we looked in more detail at methylation in oocytes. To do this, we segregated the genomes into domains of hypermethylation (HyperDomains; ≥ 75% methylation) and hypomethylation (HypoDomains; ≤ 25%), using methods we previously applied in the mouse [72]. This analysis identified 55380 HyperDomains and 54959 HypoDomains in porcine oocytes, which comprise 55% and 18% of the queried genome, respectively (Fig. 3a). On average, HyperDomains are larger than HypoDomains (median sizes 13.6 kb and 6.4 kb, respectively). Bovine oocytes, in contrast, had fewer HyperDomains but a similar proportion of HypoDomains, comprising 46% and 20% of the genome, respectively. In comparison, in human the proportion of the genome occupied by HyperDomains and HypoDomains is grossly similar to that of pig and cow with HyperDomains being prevalent over HypoDomains (53% and 33%, respectively), while in the mouse the pattern is reversed with HyperDomains occupying only 27% of the genome and HypoDomains 52% (Fig. 3a). In both mouse and human, HyperDomains are known to be predominantly associated with expressed genes [32, 72]. To test whether this applied also to bovine and porcine oocytes, we evaluated methylation levels of expressed genes from RNA-seq data we obtained from parallel pools of oocytes. Similar to what was previously reported in cow oocytes based on much sparser DNA methylation data [61], genes with high gene body methylation are more likely to be expressed, both in cow and pig (Fig. 3b). Finally, we followed the status of these domains into the preimplantation embryos. Consistent with the globally higher methylation in pig cleavage embryos, the HyperDomains were more highly methylated in pig than bovine embryos (Fig. 3c, d). We note, however, that we are not able to distinguish methylation on oocyte-derived from sperm-derived chromosomes in the embryos, and the fact that these features have a mean methylation of greater than 50% in pig embryos indicates that the residual methylation is a composite of methylation on maternal and paternal alleles. Interestingly, HyperDomains decline in methylation from the 2–4 cell stages to morula in the pig, indicating continued demethylation, but their methylation is essentially constant in the cow over the same stages (Fig. 3c, d). These findings reveal a more complex dynamic of methylation reprogramming than apparent from the global methylation figures. This complexity is further augmented by the behaviour of the oocyte HypoDomains in cleavage embryos. Specifically in the pig, there is a pronounced increase in methylation at the 8–16 cell stages (Mann-Whitney, *p* < 2.2e−16; Fig. 3c). A similar remethylation phase has been observed in human 8 cell embryos, which was found to be associated in particular with young SINEs [77]. To investigate whether there were specific sequence elements associated with regions subject to de novo methylation, we identified all 100-CpGs tiles losing or gaining methylation between the 2–4 and 8–6 cell stages, filtering for tiles changing by > 10%. In this unbiased approach, we also detected an excess of sites gaining methylation in pig (15.4%), which was approximately double that in cow (Supplementary Table 3). There was an enrichment for intergenic regions in pig and for gene bodies in cow (Supplementary Figure 4a). Specific regions showing evident gain in methylation at 8-16 cells are shown in Supplementary Figure 4b.

### Expression of genes for de novo methylation and demethylation activities

**Fig. 4:**
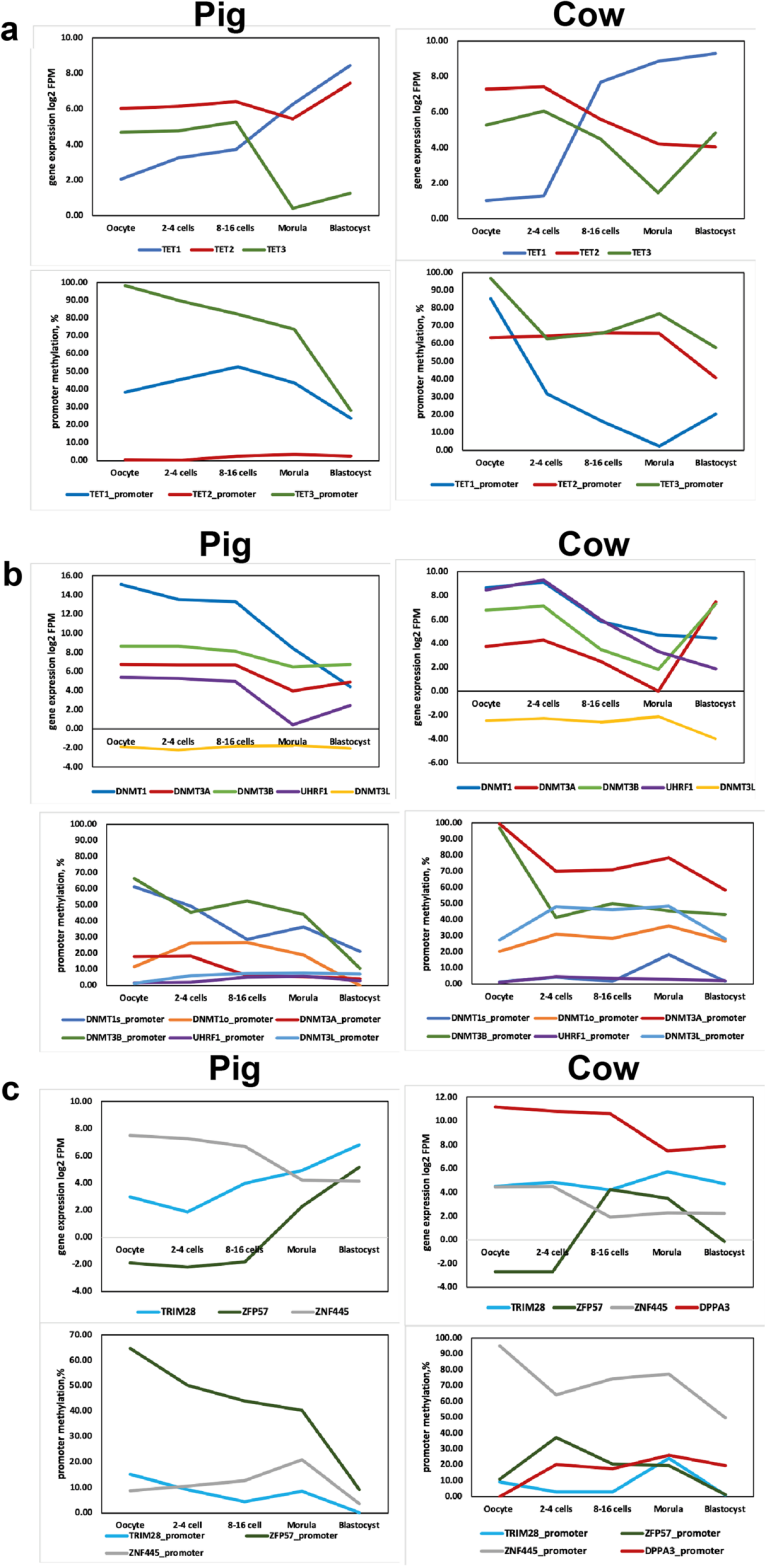
Expression and promoter methylation of genes for de novo methylation and demethylation activities. **a** TET family enzymes expression and DNA methylation at the promoters. **b** DNMTs expression and DNA methylation at the promoters. **c** Gene expression and promoter methylation for key factors known to be involved in the mouse and/or human in maintaining DNA methylation at germline differentially methylated regions (gDMRs): the zinc-finger proteins ZFP57, ZNF445, TRIM28 (KAP1) and DPPA3

We investigated the expression and methylation of the genes encoding the major activities involved in DNA methylation reprogramming as a potential explanation for the observed differences in the methylation landscapes between the species (Fig. 4). In general, the expression patterns of transcripts for TET family enzymes in the pig are consistent with previous reports using qRT-PCR, in which *TET3* predominates from the oocyte and first cleavage stage with a progressive replacement by *TET1* and *TET2* [35]; similar observations have been made in mouse [30] embryos. Bovine embryos differed in exhibiting an increase in *TET3* transcripts in blastocysts and lack of upregulation of *TET2*, whereas the dynamics of *TET1* expression were consistent with a previous report [46]. Regarding the DNMT family, transcripts for the de novo methyltransferases DNMT3A and DNMT3B as well as the maintenance methyltransferase DNMT1 and its auxiliary protein UHRF1 were readily detected in both pig and cow oocytes. But it was striking that there was no detectable expression of *DNMT3L* transcripts either in bovine or porcine oocytes or any embryo stage (Fig. 4). DNMT3L is essential as an obligate partner for DNMT3A for de novo methylation in mouse oocytes [4, 62, 63] but is not expressed in human oocytes [44]. Despite the general maintenance of global methylation levels until the morula in both pig and cow, *DNMT1* and *UHRF1* transcript levels decline markedly. In the cow, it was notable that *DNMT3A* and *DNMT3B* transcripts levels recovered very strongly from the morula to blastocyst stage (Fig. 4), which could be associated with the onset of de novo methylation observed in the cow blastocysts [12], although it is not reflected in the global genomic methylation data (Fig. 1).

Several of the *TET* and *DNMT* genes are known in the mouse and human to have alternative promoters in oocytes compared with somatic tissues, with their somatic promoters being methylated in oocytes [28, 63]. We were able to identify candidate oocyte-specific and somatic promoters for *DNMT1* in both pig and cow. In pig, RNA-seq reads demonstrate that transcripts originating from the candidate oocyte-specific promoter (which we designate *DNMT1o*) are detected in oocytes and embryos until the morula stage, but transcripts specifically mapping to the annotated, somatic promoter are absent throughout (Fig. 4; Supplementary Figure 5). In pig oocytes, the *DNMT1o* promoter is unmethylated (< 10%) and the somatic promoter methylated (~ 61%). In cow, there is evidence for transcripts initiating both from the *DNMT1o* and somatic promoters in oocytes, which are also detected in 2–4 cell embryos, after which only canonical transcripts are detected (Supplementary Figure 5). Consistent with activity of both promoters, they are both unmethylated (< 20%) in cow oocytes (Fig. 4). The annotated *TET3* promoters were highly methylated in pig and cow oocytes (Fig. 4). In the cow, the marked increase in *TET3* transcript abundance at the blastocyst stage occurs despite relative high maintenance of methylation of the somatic promoter, which might indicate activation of the gene in a subpopulation of cells that lack promoter methylation. Similarly, the pronounced upregulation of *DNMT3A* and *DNMT3B* transcripts in bovine blastocysts also occurred against relatively high levels of promoter methylation (Fig. 4).

We also evaluated expression and methylation of genes for key factors known to be involved in the mouse and/or human in maintaining DNA methylation at germline differentially methylated regions (gDMRs): the zinc-finger proteins ZFP57 and ZNF445 that have sequence-specific binding for methylated gDMRs and recruit TRIM28 (KAP1), part of the complex that mediates DNA methylation and repressive chromatin [36, 39, 41, 51, 67]. ZFP57 is a maternal effect protein for gDMR maintenance in the mouse but is not expressed in human oocytes. Strikingly, in both pig and cow *ZFP57* transcripts were undetectable in oocytes and only appeared at the 8–16 cell or morula stages; in pig, lack of expression was associated with high promoter methylation in oocytes (Fig. 4). In contrast, and similar to the situation in human [67], *ZNF445* transcripts were abundant in pig [74] and cow oocytes, suggesting that this protein substitutes for ZFP57 in the initial maintenance of gDMR methylation (Fig. 4). The detection in cow and/or pig oocytes of transcripts for TRIM28 and DPPA3, which in the mouse are maternal effect proteins for DMR maintenance (TRIM28 [41];) or for protecting the maternal genome from active demethylation (DPPA3 [42];), would be compatible with roles of these proteins also in these two species.

### Gametic DMRs and candidate imprinted genes in the pig and cow

We looked in more detail at the gametic methylation patterns as a means of understanding the nature of methylation differences and the potential for specifying imprinted genes. As a first analysis, we assigned 100-CpGs tiles as hypermethylated (≥ 75%) or hypomethylated (≤ 25%) in either gamete. Tiles hypermethylated both in sperm and oocyte generally followed the genome average in both pig and cow in relation to distribution of genomic features such as genes, CGIs and interspersed repeats (Supplementary Figure 6a,b). The same was true for sperm-specific DMRs, albeit with some enrichment for intergenic regions (Supplementary Figure 6c). In contrast, tiles hypomethylated in both gametes were very strongly enriched in CGIs and promoters, as might be expected (Supplementary Figure 6d). For oocyte-specific DMR tiles, there was also a very strong enrichment in genes and CGIs (Supplementary Figure 6e). Indeed, we identified 700 CGIs specifically methylated in pig oocytes and 1411 methylated in cow oocytes. This is comparable to mouse (1329) and human (1440) oocyte-specific methylated CGIs [32, 44].

Inspection of the oocyte-specific or sperm-specific DMRs that contained CGIs revealed that they included some of the known imprinted genes, as might be anticipated (data not shown). We further filtered the gametic DMRs for tiles that retained intermediate methylation (30–70%) through to the blastocyst stage as a means of identifying candidate imprinted genes in the pig and cow (Datasets 1 and 2). Imprinted genes have been identified in both species (listed at http://www.geneimprint.com/), but mostly as a result of candidate single gene analysis from known imprinted genes in mouse/human, or by expression analysis in parthenogenetic embryos/blastocysts. In the pig, we identified 160 oocyte-specifically methylated CGIs that retained intermediate methylation in blastocysts (Dataset 1, Maternal imp candidat CpGi targ); these included nine imprinted genes that matched the list at http://www.geneimprint.com/ or corresponded to imprinted genes in mouse and human (*DIRAS*, *HERC3*/*NAP1L5*, *IGF2R*, *INPP5F*, *MEST*, *PEG3*, *PLAGL1*, *GNAS* and *NNAT*). When we extended the analysis to any 100-CpGs tile fitting the same criteria (hypermethylated in oocytes, hypomethylated in sperm, intermediately methylated in blastocysts), there were just 183 tiles (Dataset 1, Maternal imp DMR cand Global), of which 25 coincided with imprinted gDMRs known from mouse or human; this analysis added *KCNQ1* and *PEG10*. This relatively high hit rate suggests that the approach is useful for identification of candidate imprinted genes in this species. When applying the same approach to CGIs specifically methylated in sperm, none of the 74 hits (Dataset 1, Paternal imp candidat CpGi targ) corresponded to an imprinted gene known from other species, but when the filter was relaxed for any 100-CpGs window fitting the methylation criteria, three candidate imprinted genes from the *DLK1-DIO3* domain were identified (*BEGAIN*, *MEG3*, *RTL1*) out of 374 tiles (Dataset 1, Paternal imp DMR cand Global). Therefore, for imprinted genes specifically methylated in sperm, the approach appears less successful. We used the same logic in the cow methylation datasets. Focusing on CGIs specifically methylated in oocytes, 88 met the criteria (Dataset 2, Maternal Imprint Cand CpGi targ), of which *MEST*, *SGCE* and *SNRPN* are known to be imprinted. When all 100-CpGs tiles were considered irrespective of overlap with CGIs, 1048 tiles met the criteria (Dataset 2, Maternal Imp DMR Cand Global), which included the genes *ASB4*, *BLCAP*, *B4GALNT4*, *GNASL*, *HERC3*, *MEST*, *MZF1*, *PEG10*, *TRAPPC9*, *SIM2* and *SNRPN* (representing seventeen 100-CpGs tiles or 1.52%). Therefore, it seems that the approach may be less specific in the cow, with a likely higher rate of false positives. When assessing CGIs specifically methylated in sperm, 42 were found to be intermediately methylated in blastocysts including a CGI at *IGF2* (Dataset 2, Paternal Imprint Cand CpGi targ). When all 100-CpGs windows were considered, 4004 met the criteria, including at *BEGAIN*, *IGF2*, *INS*, *RASGRF1* and *RTL1* (Dataset 2, Paternal Imp DMR cand Global), but the larger number of ‘hits’ suggests a high rate of false positives again. It is likely that the gross difference in DNA methylation levels in cow compared with pig blastocysts contributes to this apparently low specificity: with a global level of methylation of 27% in blastocysts in the cow, any imprecision in methylation level estimates from the PBAT data could place non-imprinted features into the candidate imprinted category at an inclusion threshold of ≥ 30%. As a first step towards validating the gDMRs as elements regulating potential new imprinted genes, we assessed the locations of these gDMRs in relation to genes found to be monoallelically expressed by RNA-seq analysis of placenta and fetal tissues from of a *Bos taurus taurus* x *B. t. indicus* cross [9]. Of the 45 autosomal imprinted transcripts reported by Chen et al., our DMR candidates mapped within or close to (< 50 kb) 15 known imprinted genes and close to 7 novel imprinted genes (Supplementary Table 4).

**Fig. 5:**
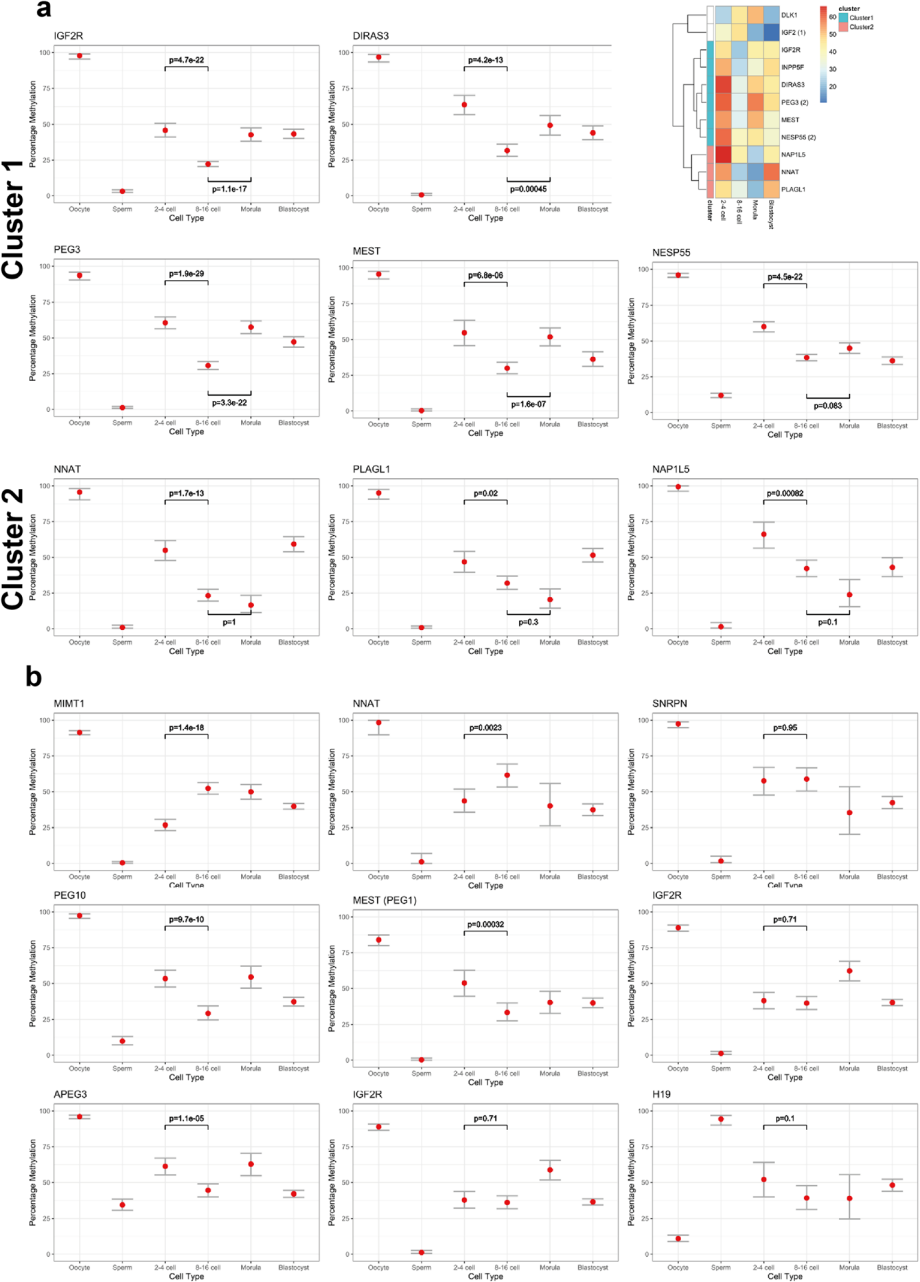
DNA methylation dynamics of candidate imprinted genes in the pig (**a**) and cow (**b**). In pig, two clusters were identified based on the time of remethylation (~ 50%): morula stage for cluster 1 and at the blastocyst stage for cluster 2

With a list of candidate imprinted gDMRs, including from confirmed imprinted genes and those with homology to human or mouse imprinted loci indicated above, we then evaluated their methylation dynamics in preimplantation embryos. For the maternally methylated gDMRs in pig, methylation levels at the 8–16 cell stages were significantly reduced compared with the 2–4 cell stages (Fig. 5a). Thereafter, two patterns were apparent, with gDMRs such as *IGF2R*, *DIRAS3*, *PEG3* and *MEST* gaining methylation to an expected ~ 50% by morula and maintaining this level in the blastocyst, whereas for gDMRs like *NNAT*, *PLAGL1* and *NAP1L5* restoration of ~ 50% methylation occurred only in the blastocyst (Fig. 5a). These patterns suggest complex remodeling of gDMR methylation over this time, which could be compatible with differences in the requirement for and timing of expression of methylation maintenance factors, such as ZFP57 or ZNF445 (Fig. 4). In bovine preimplantation embryos, in contrast, there was no consistent reduction in methylation at the 8–16 cell stages and greater apparent stability of maternal gDMR maintenance (Fig. 5b).

### Correlations between expression and promoter methylation for pluripotency and ZGA genes and hypermethylated gamete promoters

Given the prominent role during early embryo development of pluripotency genes and those related with the zygote genome activation (ZGA), we performed correlation analysis between gene expression and methylation at the promoters of these genes in our datasets. Here, again, there were some notable differences between the pig and cow. In the pig, ZGA gene promoters were relatively highly methylated in oocytes, and methylation state in oocytes showed a weak positive correlation with expression that persisted across at all embryonic stages (0.33 at the 2–4 cell stages to 0.25 in blastocysts; Supplementary Figure 7). Similar weak positive correlations were observed between methylation and expression within all stages, declining in the blastocyst. In contrast, ZGA gene promoters showed a bimodal range of methylation in cow oocytes and a complete absence of correlation between oocyte methylation and expression in cleavage embryos, or tending towards a negative correlation (Supplementary Figure 8). These general trends applied also to the smaller set of pluripotency genes (Supplementary Figure 7 and 8). Finally, we studied the gene expression dynamics following fertilisation for hypermethylated sperm and oocyte promoters that are demethylated more than 50% by the cleavage stage, as Smith et al. did with human data [65]. We did not find a significant difference in the number of demethylated gamete promoters that exhibited up or downregulation (> two-fold) in expression compared to other promoters.

## Discussion

The results of this study detail the DNA methylation dynamics during the first week of development in two major livestock species where this information was not previously available: *Sus scrofa* and *Bos taurus*. In both species, a partial picture of these events can be inferred from immunostaining data [8, 14]. More recently, reduced representation bisulfite sequencing (RRBS) [31] and single-cell genome-wide profiling [15] until the morula stage have been applied, albeit with lower CpG coverage, in the cow, but neither provide a comprehensive, genome-wide characterization of DNA methylation at the single-base level or accurate estimates of global methylation until the blastocyst stage. When comparing our results in cow and pig to those of human and mouse, we observed that the cow followed a similar pattern of demethylation from the oocyte to the 2–4 cell stages as human; however, this was not the case for pig, which is similar to mouse [21, 66]. The delay of active conversion of 5-methylcytosine (5mC) in the maternal genome to 5-hydroxymethylcytosine (5hmC) by TET3 has been explained in mouse studies by protection of the oocyte genome by STELLA/PGC7/DPPA3 [42], but the direct lack of demethylation had not been considered until now. We hypothesize that differences in the transition from maternal control of development to zygotic control, occurring at different timepoints depending on the species and connected with the pluripotency programme, could help to explain this finding. Indeed, it is accepted that in human and monkey, the major ZGA wave takes place during the 4–8 cell stages [60, 71, 73], while in the cow this happens later, at the 8–16 cell stages [68], but earlier in the pig and mouse (2 cell stages in mouse and 4 cell stages in pig) [29, 68, 75]. Therefore, the fact that ZGA in the pig and mouse occurs earlier in development could support our hypothesis. Conversely, the male genome is rapidly demethylated in both cow and pig, and this finding has also been well described in other species [21, 31, 77]. We did not observe global re-methylation peaks either in pig or cow, as described by Jiang et al. in cow [31], Zhu et al*.* in human [77] and Guo et al. in mouse [21]. These discrepancies could be explained by technical differences in the methods used in relation to the sequences covered (i.e., RRBS vs whole genome bisulfite sequencing). But we also found that a transient remethylation phase was detectable in pig embryos specifically of sequences hypomethylated in oocytes. These locus-specific fluctuations occurred in the presence of relatively constant levels of *Dnmt3A*/*3B* transcripts during first cleavage divisions. The dynamics of DNMT expression in oocytes and early embryos have been characterised in mouse, bovine, rhesus monkey and humans and shown to exhibit differences between species (reviewed by [70]). We detected progressive reduction in expression and promoter methylation of DNMT1 in the pig from the oocyte to the blastocyst stage. The observed decrease in *DNMT1* transcripts could represent the degradation of oocyte-derived mRNA. In our RNA-seq data, we detected an additional *DNMT1* transcript with a transcriptional start site upstream of the canonical promoter in both pig and cow oocytes; this could represent *DNMT1o*, an oocyte-specific transcript earlier described in human [24] and mouse [40], but its existence in cow has been considered unlikely [56]. From RNA-seq data, it appears that *DNMT1* transcripts identified in the oocyte and early embryo in pig originated from this alternative promoter, after which *DNMT1* was expressed from the somatic promoter. In the cow, in contrast, both *DNMT1o* and somatic-form transcripts were present in the oocyte and 2–4 cell embryos, indicating a striking difference in the regulation between the species. The switch from the oocyte to somatic isoforms may be important for sub-cellular localisation of the corresponding proteins. In cow, as in the mouse, DNMT1 is found in the cytoplasm in zygotes but not in 8–16 cell embryos where it was mostly nuclear [37]. The switch to somatic form of DNMT1 in cow at 8–16 cell stages could be important before de novo methylation by DNMT3A and DNMT3B initiates at the blastocyst stage. DNMT3A and DNMT3B showed a significant increase in expression from the morula to the blastocyst stage in the cow; there was no comparable increase in the pig. In previous studies, it was suggested that de novo methylation in the cow starts from the 8–16 cell stages [12]. This could account for why the methylation level observed at the blastocyst stage in the cow (around 27%) did not drop to values as low as those observed in the pig (around 13%). In comparison, results from immunostaining in the pig indicated that methylation reaches a minimum at the late blastocyst stage [13] and, according to our RNA-seq data, *DNMT3A* and *DNMT3B* expression remained low at this time. Low levels of DNMT3A and DNMT3B in pig blastocysts have also been observed by others [76]. We suggest that unlike cow, de novo methylation does not start until later embryonic stages in pig, possibly until blastocyst expansion. The increasing expression of TET1 and TET2 enzymes through these first days of development shown in our data could also explain the extremely low levels of methylation in pig blastocysts. High levels of *TET3* transcripts in both cow and pig from oocyte to the 8–16 cell stages likely reflect its role in active demethylation of the paternal genome, as has been proposed from extensive mouse studies [25]. However, the gene expression profile and promoter methylation of components of the DNA methylation machinery cannot fully explain the variation in global methylation landscape or in imprinted gene reprogramming amongst the species. It is possible that other epigenetic mechanisms, such as post-translational modifications, are involved in the process of gene expression regulation, acting specifically at promoters devoid of CGIs, for example H3K9me2/3 marking inactive heterochromatin in CGI-poor promoters [2].

It is generally accepted, from murine and human data, that DNA methylation marks at the DMRs of imprinted genes are established in the parental germline and robustly maintained through early development, by the action of a complex set of factors including DNMT1, ZFP57 and/or ZNF445 and KAP1/TRIM28 [10, 26, 33, 66]. However, our data suggest a greater dynamic of gDMR methylation, and few appear to maintain the expected 50% methylation from the 2–4 cell stages to blastocyst stage; moreover, different patterns were apparent amongst gDMRs, particularly in pig. These results are compatible with previous suggestions [43] that DNA methylation status of imprinted gene DMRs during the first days of development might be variable and could require an active period of imprint stabilization until the blastocyst stage [69]. It is possible that the differences in methylation dynamics of various gDMRs reflect differences in dependence on factors such as ZFP57 and ZNF445 in methylation maintenance [67], and our observation that the transcripts for these two factors have distinct expression patterns from the oocyte to blastocyst in both species may be important in this context.

We used our methylation datasets to identify CGIs (or 100-CpG windows) that have gametic and embryo methylation patterns consistent with parent-of-origin monoallelic methylation as a means towards discovering potential new imprinted sequences, as has been done in other species [32, 44]. This approach appears more promising in pig than in cow, judging by the greater proportion of known imprinted genes amongst the candidates DMRs in pig and the much higher number of candidates in cow, probably on account of the higher genome-wide methylation in cow blastocysts. Nevertheless, 7 of our candidates mapped close to novel imprinted transcripts identified in a recent study that employed RNA-seq in fetal bovine tissues [9] . It should be noted that monoallelic methylation in the blastocyst is not a guarantee of persistence of imprinting at later stages, as many CGIs that retain parent-of-origin methylation in mouse blastocysts become biallelically methylated or biallelically unmethylated post implantation, a phenomenon termed ‘transient imprinting’ [49]. This is not to say that transient imprints are inconsequential, as long-lasting imprinted expression states can be set up with physiological significance although the initiating methylation mark is lost, as in the case of the mouse *Zdbf2* locus [16, 20]. In addition, in human, there is a greater persistence of DMRs in the placenta than embryo proper [23, 58], which might indicate a greater role for non-classical imprinted genes in this tissue. The full significance of gametic methylation beyond classical imprinted genes remains to be elucidated.

In terms of the functions of imprinted genes, both PEG10 and NNAT proteins are highly expressed in placenta and play crucial roles during early development, being associated with normal formation of the placenta itself [45] (*PEG10*) and to the formation of nervous system Ca^2+^ signalling, glucose transport, insulin secretion and inflammation [48] (*NNAT*). Transcripts from *GNAS* complex locus, on the other side, are involved in different signal transduction pathways and a variety of cellular responses, having being also associated with intrauterine growth retardation and thus small size for gestational age [54]. The precise physiological significance of these observations remains to be further investigated.

It should be noted that the embryos analysed in this study were obtained after in vitro maturation of oocytes, in vitro fertilisation and embryo culture. The potential for these manipulations and culture media to impair normal DNA methylation events in oocytes and maintenance in cleavage embryos has been much discussed [5–7, 74]. At this time, it appears that interventions required to obtain mature oocytes have limited impact on the establishment of normal DNA methylation patterns in oocytes themselves (e.g., [57]), but there is more evidence that they, or embryo culture, can lead to some compromise in methylation reprogramming or maintenance of imprinted methylation in preimplantation embryos [5–7, 9, 17, 18]. Therefore, the methylation patterns of purely in vivo derived gametes and embryos could differ in some details from the results presented here. Similarly, it should be noted that porcine oocytes were obtained from prepubertal rather than adult sows. In the mouse, it has been described that although the DNA methylation patterns are highly similar, there are a limited number of discrete DNA methylation differences in oocytes collected from immature compared with mature females [57]; a similar effect could apply in pig.

In conclusion, we provide a detailed comparative evaluation of the DNA methylation patterns of oocyte and sperm, and the post-fertilisation dynamics of methylation, in the cow and pig. Our results indicate differences between these two major livestock species, and differences in several respects from the pattern observed in mouse, which has been the model organism of choice to now. These differences extend to the timing and extent of methylation reprogramming and the expression pattern of the key methylation and demethylation activities. Moreover, our data support the hypothesis we previously suggested [5–7] about a general uncoupling between DNA methylation and gene expression during demethylation of gametes at preimplantation development. This also applies to imprinted genes. In many respects, the expression patterns of DNMTs as well as the zinc-finger proteins ZFP57 and ZNF445 critical for maintenance of DNA methylation at imprinted genes in these species mirror those of human gametes and embryos more closely than the mouse, suggesting that they could provide a more faithful model for human for the regulation of epigenetic reprogramming. These data also provide a valuable reference against which to assess the epigenetic fidelity of ART.

## Materials and methods

### Collection of gametes and embryo samples

#### In vitro maturation (IVM) for collection of pig oocyte samples

The in vitro maturation (IVM) was performed as previously described [11]. Briefly, within 30 min of slaughter, ovaries from pre-pubertal Landrace-Large-White gilts were transported to the laboratory in saline containing 100 mg mL^−1^ kanamycin sulphate at 38 °C, washed once in 0.04% cetrimide solution and then twice in saline. Cumulus cell-oocyte complexes (COCs) were collected by aspiration from antral follicles (3 to 6 mm diameter), washed twice with Dulbecco’s PBS supplemented with 1 mg mL^−1^ PVA, then washed twice more in maturation medium previously equilibrated for a minimum of 3 h at 38.5 °C under 5% CO_2_ in air. Maturation medium NCSU-37 [47] supplemented with 0.57 mM cysteine, 1 mM dibutyryl cAMP, 5 mg mL^−1^ insulin, 50 mM β-mercaptoethanol, 10 IU mL^−1^ equine chorionic gonadotropin (eCG; Folligon; Intervet International BV, Boxmeer, Holland), 10 IU/mL hCG (VeterinCorion; DivasaFarmavic, Barcelona, Spain) and 10% porcine follicular fluid (v/v) was used.

Groups of 50–55 COCs with complete and dense cumulus oophorus were cultured in 500 μL maturation medium for 22 h at 38.5 °C and 5% CO_2_ in air saturated of humidity. After culture, COCs were washed twice in fresh maturation medium without dibutyryl cAMP, eCG and hCG and cultured for an additional 20–22 h [19].

#### In vitro fertilisation (IVF) and embryo culture (EC) for collection of pig embryo samples

After 42–44 h of maturation, cumulus cells were removed by pipetting, and groups of 45 to 50 denuded oocytes were transferred into each well of a 4-well multidish containing 250 μL TALP medium [52] previously equilibrated at 38.5 °C under 5% CO_2_ in air. Semen samples were collected by the gloved hand method from fertility-tested boars (1–2 years old) and immediately transported to the laboratory. Sperm were processed by the swim-up method using NaturARTs® PIG sperm swim-up medium (Embryocloud, Murcia, Spain) as previously described [5–7]. Briefly, the swim-up medium was supplemented with bovine serum albumin 3 mg/mL (BSA-FAF), and 1 mL of ejaculated spermatozoa was lay below 1 mL of NaturARTs® PIG sperm swim-up medium at the bottom of a conical tube. After 20 min of incubation at 37 °C (with the tube at a 45 ° angle), 0.75 mL from the top of the tube was aspirated and concentration adjusted to 10^5^ cells/mL in TALP medium for insemination of the IVF dishes with 250 μL of this suspension. Spermatozoa and oocytes were cocultured at 38.5 °C under 5% CO_2_ in air saturated of humidity and 18 h post insemination (hpi); the putative zygotes were washed and transferred to embryo culture medium. For embryo culture, NCSU-23 media [47] supplemented with sodium lactate (5 mM), pyruvate (0.5 mM), non-essential amino acids and 0.4% BSA-FAF (NCSU-23A, for the 18 to 48 hpi) or NCSU-23 supplemented with glucose (5.5 mM), essential, non-essential amino acids and 0.4% BSA-FAF (NCSU-23B, for 48–168 hpi) were used. At 48, 72, 120 and 168 hpi, pools of forty 2–4 cell embryos, twenty 8–16 cell embryos, ten morula and single expanded blastocyst were selected, washed in PBS and ZP digested by 0.5% w/v pronase in PBS. Finally, embryos were washed three times in PBS and snap frozen in liquid nitrogen until further processing.

#### Ovum pick up for cow oocyte collection

Non-lactating and non-pregnant cows (*n* = 3) located at the Farm Dairy facilities of the University of Murcia, Spain, were used as oocyte donors. Ultrasound evaluation was performed in order to assess the size of follicle(s) or to count the number of puncturable follicles. Ovum pick up was performed on ≥ 15 mm of diameter follicles. The system used for follicle aspiration was a Falco-Vet ultrasound with a 10R transvaginal probe at 7.5 MHz (Esaote, Genova, Italy). Cows were given xylazine (Nerfasin®, Fatro, Barcelona, Spain—0.25 mL/100 kg weight, IM), carprofen (Carprosan®, Fatro, Barcelona, Spain—1.4 mg/kg weight, SC) and lidocaine (Anesvet, Ovejero, León, Spain—2%, 5 mL, epidural). For oocyte retrieval, the aspiration pump (Aspirator 3—Labotect, Göttingen, Germany) applied a pressure of 70 mm Hg and 20 mL/min, and the system included the punction-needle (with a disposable 18G needle) connected via a sterile tube to a Falcon tube. The medium used to collect oocytes was Dulbecco’s phosphate buffered saline, supplemented with 1% (v/v) Fetal bovine serum and 2.2 UI/mL heparin, pre-heated at 38 °C. After aspiration, oocytes were collected under a stereomicroscope (Nikon SMZ 10A, Japan) and washed twice in Dulbecco’s phosphate buffered (without calcium or magnesium, PBS) supplemented with 0.5% polyvinyl alcohol (wt/v). Cumulus cells were removed with gentle pipetting, and vortex was used when necessary, as well as hyaluronidase (0.2% in PBS). Zona pellucida was removed using pronase (0.5% in PBS). Oocytes were put in 5 μL of RLT buffer (Qiagen, Germany), immediately frozen in liquid nitrogen and stored at – 80 °C.

#### IVM and IVF for collection of bovine embryo samples

Immature cumulus-oocyte complexes (COCs) were obtained by aspirating follicles (2–8 mm) from the ovaries of matured heifers and cows collected at slaughter from local abattoirs. A total of 1425 COCs with homogenous cytoplasm and intact cumulus cells were matured in four-well dishes (Nunc, Roskilde, Denmark) in 500 μL of TCM-199 maturation medium, supplemented with 10% (v/v) fetal calf serum (FCS) and 10 ng/mL epidermal growth factor (EGF) in 4 well dishes in groups of 50 COCs per well at 38.5 °C under an atmosphere of 5% CO2 in air with maximum humidity [55].

After 24 h of maturation, a representative number of the in vitro matured COCs were suspended in 100 μL of PBS without calcium or magnesium supplemented with 1% BSA and their cumulus cells (CCs) completely removed by pipetting. Only oocytes presenting polar body were selected and snap frozen in pools of 100 in LN_2_ and stored at – 80 °C until use (*n* = 2).

The remaining matured oocytes were in vitro fertilized (IVF). IVF was performed as described previously [38]. Briefly, frozen semen straws from an Asturian Valley bull (ASEAVA, Asturias, Spain) previously tested for IVF were treated with Bovipure™ (Nidacon, Sweden). Sperm concentration was determined and adjusted to a final concentration of 1 × 10^6^ spermatozoa/mL. Gametes were co-incubated for 18–20 h in 500 mL fertilisation media (Tyrode’s medium with 25 mM bicarbonate, 22 mM sodium lactate, 1 mM sodium pyruvate and 6 mg/mL fatty acid-free BSA supplemented with 10 mg/mL heparin sodium salt; Calbiochem) in a four-well dish in groups of 50 COCs per well under an atmosphere of 5% CO2 in air, with maximum humidity at 38.5 °C.

After 18–20 h post-insemination (hpi), presumptive zygotes were denuded of cumulus cells by vortexing and then cultured in groups of 25 in 25 μL droplets of synthetic oviductal fluid (SOF) [27] with 4.2 mM sodium lactate, 0.73 mM sodium pyruvate, 30 μL/mL BME amino acids, 10 μL/mL minimum essential medium (MEM) amino acids and 1 mg/mL phenol red, supplemented with 3 mg/mL BSA, under mineral oil at 38.5 °C under an atmosphere of 5% CO_2_, 5% O_2_ and 90% N_2_ with maximum humidity.

At 33, 54, 132 and 168 hpi, pools of fifty 2 cell embryos, thirty 8 to 16 cell embryos, ten morulaes and individual blastocysts were selected respectively, vortexed in PBS for 3 min and were then treated with 0.5% w/v pronase in PBS to digest the zona pellucida. They were finally washed three times in PBS and snap frozen in LN_2_ and stored at − 80 °C until use (*n* = 2 per embryo stage).

#### RNA preparation and RNA-seq

ARCTURUS® PicoPure® RNA Isolation Kit (KIT0204, Life Technologies) was used to extract the RNA from oocytes, embryo pools or from individual blastocysts. RNA-seq libraries were generated using Ovation RNA-Seq System V2 (NuGEN, Cat. 7102-08) for low amount of starting material and further amplified with NEB Next DNA Library Prep Master Mix for Illumina for 8 PCR cycles (NEB, Cat. E6040S). All steps for RNA extraction and library preparation were performed according to manufacture guidelines. iPCRTag reverse primer with individual index was used to generate one biological replicate from each condition. One hundred base pairs single end reads were sequenced on Illumina HiSeq 1000, and sequencing data were bioinformatically processed.

#### Transcriptome analysis

Raw sequence reads were trimmed using Trim Galore to remove adapter contamination and reads with poor quality defined by low PHRED score. Data were mapped to assembly UMD3.1 (cow) or Sus scrofa 11.1 (pig); hisat algorithm was used to process the reads, and data were visualised with the Seqmonk software package (v.1.41; Babraham Institute). Only reads with high PHRED score were used in the analysis. Data were passed through an additional filter of having mapped quality score 20 or more before the processing.

Annotated mRNA features were quantitated as log2FPM (fragments per million reads of library) to obtain respective expression values.

Additionally, RNA seq data were used to look for alternative promoters; oocyte specific isoforms of Dnmt1 transcripts (Dnmt1o) were identified for both porcine and bovine embryos as well as oocyte specific TET2 transcript was detected in bovine embryos, and genomic regions 1500 bp upstream and 500 bp downstream of these alternative transcripts were used as promoters.

#### DNA library preparation based on post-bisulfite adapter tagging

An adaptation of whole genome bisulfite sequencing that involves post-bisulfite adapter tagging (PBAT) was used to analyse the methylome of germ cells and early embryos at single-base resolution on a genome-wide scale, as previously performed [6].

With the exception of bovine oocytes, at least two replicate PBAT libraries were generated from each stage, each comprising the equivalents of ~ 80–150 cells per sample. In the case of the blastocyst stage, libraries were generated from single whole blastocysts. For porcine oocytes, PBAT was conducted on metaphase-II (MII)-stage oocytes obtained after in vitro maturation (IVM). Bovine oocytes were obtained by ovum pick-up after natural ovulation or hormonal stimulation and were processed for single-cell PBAT using the protocol described by [64] in this case, data from 28 individual oocyte libraries were merged for analysis.

For sperm methylome, bull and boar sperm DNA was extracted from ejaculates of two biological individuals. Cells were lysed in the lysis buffer containing 10% SDS with proteinase K, 20 mM Tris, 10 mM DTT, 10 mM EDTA, 150 mM NaCl and 10 mM KCl for 2 h at 50 C. Further, DNA was purified with phenol to chloroform at 1:1 ratio and precipitated with equal volume of isopropanol. DNA pellet was washed with 70% ethanol, air dried and dissolved in EB buffer. DNA concentration was measured using Nanodrop, and 10 ng was used for bisulfite conversion and PBAT library preparation as described below. For other biological timepoints, pools of oocytes (100), 2 cell embryos (40), 8 cell embryos (30), morulae (10) or individual blastocysts were lysed for 1 h in 1% SDS with proteinase K, and lysates were directly treated with bisulfite reagent using the Imprint DNA modification kit (Sigma, MOD50). DNA was eluted in EB buffer, and one round of first strand synthesis was performed using a biotinylated oligo 1 (5-[Btn] CTACACGACGCTCTTCCGATCTNNNNNNNNN-3). Samples were further treated with Exonuclease I, washed and eluted in 10 mM Tris-Cl and incubated with washed M-280 Streptavidin Dynabeads (Life Technologies) to pull down the biotinilated fraction of DNA. Second strand synthesis was performed using oligo 2 (5′-TGCTGAACCGCTCTTCCGATCTNNNNNNNNN-3′), and samples were amplified for 12 PCR cycles using indexed iPCRTag reverse primer [50] with KAPA HiFiHotStart DNA Polymerase (KAPA Biosystems) and purified using 0.8 × AgencourtAmpure XP beads (Beckman Coulter). Libraries were assessed for quality and quantity using high-sensitivity DNA chips on the Agilent Bioanalyzer. Two biological replicates were generated for each of biological timepoint and prepared for 100 bp single-end sequencing on Illumina HiSeq 1000 and sequenced at 4 samples per lane.

#### Methylome analysis

Library sequence reads were mapped to the pig (genomic assembly Sus scrofa 11.1) and cow (genomic assembly UMD 3.1) genomes using the Bismark software (v.0.19; Babraham Institute). DNA methylation analysis was done using the the SeqMonk software package (v.1.41; Babraham Institute).

For the unbiased analysis of studied genomes, 100 CpGs tiles were defined using CpG occurrences annotation track for each of the specie studied. Then, the bisulfite quantitation pipeline was run over existing tiles, 1 minimum count to include position and 5 minimum observations to include feature (100 CpGs tile). Only informative tiles were included in analysis, and tiles without data in all studied biological timepoints were removed. For this, the filter on values for individual tiles was applied, where values had to have value above 0 for exactly 6 of the 6 selected data stores, representing sperm, oocyte, 2–4 cells, 8–16 cells, morula and blastocyst timepoints.

To search for the molecular mechanisms regulating the methylation dynamics observed, the study analysed the methylation and expression status at specific families of genes involved in reprogramming. This was done using SeqMonk platform and running a targeted analysis against promoters of the genes using bisulfite methylation pipeline. Computational approach was used to define gene promoters as genomic regions of 1500 bp upstream and 500 bp downstream of transcriptional start sites. CpG coverage for promoters is supplied in Supplementary table 5. Methylation values were calculated in percentage of methylated CpGs/unmethylated CpGs per feature.

Additionally, targeted analysis of methylation and gene expression over various genomic features, including imprinted genes, was performed. First, specific annotations for the pig and cow species were built manually, looking for the locations of the genes showed at http://www.geneimprint.com/ for each species at www.ncbi.nlm.nih.gov/gene. Second, their methylation (based on at least 10 methylation calls per feature) and gene expression values were obtained. Third, germline specific DMRs were searched: for this, 100 CpGs tiles were used and filtered those with the methylation value above 75% methylation in either sperm or oocyte to obtain hypermethylated DMRs. Then, a second filter was applied to these DMRs to obtain only those that were specific to one of the germline only, i.e. methylation > 75% in oocytes and < 25% in sperm, and vice versa. For analysis of the DNA methylation dynamics of candidate imprinted genes, the clustering was performed using a PCA and the separation based on the first 2 components. Chi-square test was used to obtain the indicated *p* values. Standard programmes in the R Studio were used in both cases.

For the targeted analysis of other genomic features such as CGIs (CpG islands), intergenic regions, hypo- and hypermethylated oocyte domains (Hypo- and HyperDomains) filter of 1 count per position (CpG) and 10 observations (10 cytosines) per feature were used. For repetitive elements, 1 count per position and 5 observations per feature were selected. Methylation values were calculated as indicated above for reprogramming genes.

Finally, the possible correlation between the methylation values obtained at each stage and the gene expression values in the corresponding CpGi of the gene promoters was investigated. For this analysis, the steps described by Smith et al. in human [65] were followed. Briefly, the correlations were calculated with the statistical program R. For each graph, a table was generated with the expression and methylation values of the genes, where the rows will be the genes in question and the columns the different stages of development, both in methylation and expression. That table was used to calculate Pearson’s correlation, and the plot was made with the ggcorrplot package. This analysis included the genes listed in the Supplementary Table 6.

## Supplementary information

**Additional file 1: Supplementary Table 1.** Sequencing outputs for (a) PBAT or scBS-seq libraries from bovine samples; and (b) PBAT libraries from porcine samples. **Supplementary Table 2.** Sequencing output for RNA-seq libraries from porcine and bovine samples. **Supplementary Table 3.** 100-CpG tiles losing or gaining methylation between the 2-4 and 8-6 cell stages, filtering for tiles changing by >10%. **Supplementary Table 4.** Location of candidate gDMRs with respect to bovine imprinted genes identified by RNA-seq [9]. **Supplementary Table 5.** Promoter methylation and CpG coverage of DNMT, TET and related genes in pig and cow PBAT/scBS-seq datasets. **Supplementary Table 6.** Lists of pig and cow pluripotency and zygotic genome activation genes.

**Additional file 2: Supplementary Figure 1.** Principal Component Analysis (PCA) of replicate PBAT datasets from porcine and bovine gametes and preimplantation embryos, and merged scBS-seq datasets from bovine oocytes. **Supplementary Figure 2.** CpG coverage rates of merged PBAT or scBS-seq datasets from porcine and bovine gametes and preimplantation embryos. **Supplementary Figure 3.** The CpG methylation profiles from gametes to blastocyst in pig and cow; additional screenshots to accompany Figure 2a and b. **Supplementary Figure 4.** Characteristics of regions gaining DNA methylation between the 2-4 and 8-16 cells stages: enrichment analysis for various genomic features; screenshots of representative regions. **Supplementary Figure 5.** Detection of DNMT1 transcripts originating from oocyte-specific or somatic promoters: screenshots of RNA-seq alignments and intron-spanning alignments. **Supplementary Figure 6.** Properties of genomic elements differentially methylated in oocytes and sperm in pig and cow. **Supplementary Figure 7.** Correlations between expression and promoter methylation for pluripotency and zygotic genome activation genes in gametes and preimplantation porcine embryos. **Supplementary Figure 8.** Correlations between expression and promoter methylation for pluripotency and zygotic genome activation genes in gametes and preimplantation bovine embryos.

## Data Availability

Data generated during this study are included in this published article, and its supplementary information files or available from the corresponding author on reasonable request.

## Abbreviations

ART: Assisted reproductive technologies
BSA-FAF: Bovine serum albumin fatty acid free
cAMP: *Cyclic adenosine monophosphate*
CGIs: CpG islands
COCs: Cumulus cell-oocyte complexes
CpGi: CpG islands
DMR: Differentially methylated region
DNMT: DNA methyl transferase
DOHAD: *Developmental origins of health and disease*
EC: Embryo culture
eCG: Equine chorionic gonadotropin
EDTA: Ethylenediaminetetraacetic acid
EGF: Epidermal growth factor
FCS: Fetal calf serum
gDMR: Germline differentially methylated regions
H3K9me2/3: Histone 3 lysine 9 di- and tri-methylation
hCG: Human chorionic gonadotropin
hpi: Hours post-insemination
IVF: In vitro fertilisation
IVM: In vitro maturation
LINEs: Long interspersed nuclear elements
MEM: Minimum essential medium
MII: Metaphase-II
PBAT: Post-bisulfite adaptor tagging
PBS: Phosphate buffered saline
PCA: Principal component analysis
PVA: Polyvinyl alcohol
qRT-PCR: Quantitative real time polimerase chain reaction
RRBS: Representation bisulfite sequencing
scPBAT: Single cell post-bisulfite adaptor tagging
SDS: *Sodium dodecyl sulphate*
SINEs: Short interspersed nuclear elements
SOF: Synthetic oviductal fluid
TET: Ten-eleven translocation
ZFP: Zinc-finger protein
ZGA: Zygote genome activation
