# Correction to: DNA methylation changes during preimplantation development reveal interspecies differences and reprogramming events at imprinted genes

**DOI:** 10.1186/s13148-020-00887-5

**Published:** 2020-06-29

**Authors:** Elena Ivanova, Sebastian Canovas, Soledad Garcia-Martínez, Raquel Romar, Jordana S. Lopes, Dimitrios Rizos, Maria J. Sanchez-Calabuig, Felix Krueger, Simon Andrews, Fernando Perez-Sanz, Gavin Kelsey, Pilar Coy

**Affiliations:** 1 Epigenetics Programme, The Babraham Institute, Cambridge, CB22 3AT UK; 2 Physiology of Reproduction Group, Departamento de Fisiología, Universidad de Murcia, Campus Mare Nostrum, 30100 Murcia, Spain; 3 Instituto Murciano de Investigación Biosanitaria, IMIB-Arrixaca-UMU, 30120 Murcia, Spain; 4 Departamento Reproducción Animal, INIA, Madrid, Spain; 5 Bioinformatics Group, The Babraham Institute, Cambridge, CB22 3AT UK; 6 Centre for Trophoblast Research, University of Cambridge, Cambridge, CB2 3EG UK

**Correction to: Clin Epigenet 12, 64 (2020)**

**https://doi.org/10.1186/s13148-020-00857-x**

After the publication of the original article [1], an error was identified in the ‘Availability of data and materials’ section.

The sentence currently reads:

Data generated during this study are included in this published article, and its supplementary information files or available from the corresponding author on reasonable request.

The sentence should read:

Data generated during this study are included in this published article and its supplementary information files and have been deposited on GEO, accession number GSE143850.
