# Supplementary material for: DNA methylation changes during preimplantation development reveal inter-species differences and reprogramming events at imprinted genes
Source: Clin Epigenetics. 2020 May 11;12:64. doi: 10.1186/s13148-020-00857-x (PMC7216732; doi:10.1186/s13148-020-00857-x)
Supplement: Supplementary file 1 — Additional file 1: Supplementary Table 1. Sequencing outputs for (a) PBAT or scBS-seq libraries from bovine samples; and (b) PBAT libraries from porcine samples. Supplementary Table 2. Sequencing output for RNA-seq libraries from porcine and bovine samples. Supplementary Table 3. 100-CpG tiles losing or gaining methylation between the 2-4 and 8-6 cell stages, filtering for tiles changing by >10%. Supplementary Table 4. Location of candidate gDMRs with respect to bovine imprinted genes identified by RNA-seq [9]. Supplementary Table 5. Promoter methylation and CpG coverage of DNMT, TET and related genes in pig and cow PBAT/scBS-seq datasets. Supplementary Table 6. Lists of pig and cow pluripotency and zygotic genome activation genes. [file 13148_2020_857_MOESM1_ESM.pdf]

Supplementary Table 1a. Sequencing output for DNA libraries from bovine samples.

Sample	Total reads	Aligned reads	Uniquely aligned reads	Duplication rate, %	Number of unique CpG covered	% methylated CpG	% methylated CHG	% methylated CHH
Sperm bull 1	69,090,542	39,378,678	35,418,569	10,1	18010659	78,0	0,7	0,7
Sperm bull 2	48,530,352	28,559,871	26,112,115	8,6	15694987	73,9	0,7	0,7
Oocyte	259,049,767	96,826,156	31,538,826	32,6	16580914	44,0	5,3	7,6
2-cell_1	43,533,718	26,259,403	18,347,564	30,1	12621399	41,9	3,0	1,7
2-cell_2	42,077,721	26,650,377	17,396,648	34,7	11910607	43,2	3,0	1,8
8-cell_2	56,735,461	32,266,646	27,016,443	16.1	15727281	41,0	1,6	1,1
8-cell_2	11,473,611	7,055,457	5,371,611	23,9	14841042	47,5	2,0	1,4
Morula_1	39,270,891	24,330,027	19,383,765	20,3	11881737	41,0	1,4	1,2
Morula_2	27,448,301	17,515,637	14,819,766	15,4	10488824	46,4	1,8	1,5
Blastocyst_1	133,179,441	78,091,499	52,593,450	32,7	20659761	26,9	0,6	0,5
Blastocyst_2	113,715,292	66,115,083	46,088,061	30,3	19809100	27,7	0,6	0,5

Supplementary Table 1b. Sequencing output for libraries from porcine samples.

Sample	Total reads	Aligned reads	Uniquely aligned reads	Duplication rate, %	Number of unique CpG covered	% methylated CpG	% methylated CHG	% methylated CHH
Sperm_1	73,055,325	40,551,088	35,207,833	13,2	21,521,487	83,7	0,7	0,7
Sperm_2	66,503,687	44,345,269	40,960,281	7,6	21,486,398	82,6	0,8	0,8
Oocyte_1	63,289,771	49,091,049	14,690,679	70,1	11,278,754	53,1	2,7	2,9
Oocyte_2	15,835,299	10,535,838	5,878,139	44,2	6,317,827	50,9	2,9	3,3
Oocyte_3	15,589,715	10,064,346	5,298,908	47,3	5,759,096	51,6	2,8	3,2
Oocyte_4	16,578,439	10,025,265	5,350,633	46,6	5,867,475	54,1	3,4	3,7
Oocyte_5	13,580,648	8,856,180	4,542,910	48,7	5,142,056	51,6	3,0	3,5
2-cell_1	36,787,385	27,553,946	17,378,864	36,9	13,328,572	52,3	1,2	1,1
2-cell_2	31,993,729	21,967,327	16,108,398	26,7	13,127,385	55,2	1,8	1,6
8-cell_1	36,612,497	23,626,361	14,858,537	37,1	13,491,700	54,2	1,9	1,8
8-cell_2	35,614,964	21,532,427	15,469,723	28,2	13,976,045	52	1,3	1,3
8-cell_3	37,858,003	23,962,428	18,082,340	24,5	15,307,760	49,5	1,2	1,1
Morula_1	57,620,949	45,442,387	13,067,608	71,2	9,313,262	51,3	1,0	0,8
Morula_2	40,396,985	28,333,818	19,041,543	32,8	14,278,021	45,1	1,5	1,3
Blastocyst_1	77,321,112	50,114,529	29,093,731	41,9	18,058,469	15	1,1	1
Blastocyst_2	57,763,014	40,461,906	20,899,272	48,3	14,790,384	12,5	1,4	1,3

Supplementary Table 2. Sequencing output for RNA-seq libraries from bovine and porcine samples

Sample	Total number of sequenced reads	Number of genes covered per library, FPM
Pig oocyte	9599889	10051
Pig 2-4 cell	11861244	10546
Pig 8-16 cell	10775692	11501
Pig morula	10542646	11218
Pig blastocyst	13347163	10770
Cow oocyte	9257323	10529
Cow 2-4 cell	9099864	10828
Cow 8-16 cell	10549909	11074
Cow morula	6579584	11216
Cow blastocyst	35090754	10908

The number of total reads obtained per library supplied in the table only includes reads with high base call PHRED scores

Supplementary Table 3.
100-CpG tiles losing or gaining methylation between the 2-4 and 8-6 cell stages, filtering for tiles changing by >10%

Specie	Total tiles	Gain (number/%)	Loss (number/%)
**Pig**	**253122**	**39105 (15.4%)**	**40696 (16.1%)**
**Cow**	**256422**	**18941 (7.4%)**	**42458 (16.6%)**

Supplementary Table 4. Location of candidate gDMRs with respect to bovine imprinted genes identified by RNA-seq [ref. 9].

Gene ID	Gene symbol	Our data Imprinted Y or N	Located within Kb
ENSBTAG00000007975	*ALDH8A1*	*N*	
ENSBTAG00000009725	*AOX1*	*N*	
ENSBTAG00000019616	*APCS*	*N*	
ENSBTAG00000036127	*AS3MT*	*N*	
ENSBTAG00000017716	*BEGAIN*	*Y*	*inside the gene (from the candidate list)*
ENSBTAG00000008612	*C1R*	*N*	
ENSBTAG00000004840	*C1S*	*N*	
ENSBTAG00000006136	*CDA*	*Y(?)*	*29 Kb (from the candidate list)*
ENSBTAG00000031184	*CDKN1C*	*Y(?)*	*17Kb (from the candidate list)*
Unannotated*	*COPG2IT1*	*Y(?)*	*19Kb from MEST*
ENSBTAG00000012182	*DIRAS3*	*N*	
ENSBTAG00000037899	*DLK1*	*Y(?)*	*8Kb (from the candidate list)*
ENSBTAG00000017475	*GNAS*	*Y(?)*	*inside the gene (from GNAS1 and GNAS2 DMRs)*
Unannotated*	*GNASXL*	*Y*	*overlaps with GNAS(1) DMR*
ENSBTAG00000013066	*IGF2*	*Y*	*inside the gene (from the candidate list)*
ENSBTAG00000002402	*IGF2R*	*Y*	*inside the gene (from IGF2R(2) DMR)*
ENSBTAG00000021801	*INPP5F*	*N*	
ENSBTAG00000016165	*KRT7*	*N*	
ENSBTAG00000045998	*MAGEL2*	*N*	
ENSBTAG00000010128	*NAP1L5*	*Y*	*inside the gene (from Imprinting cow SCB 2)*
ENSBTAG00000019600	*OOEP*	*N*	
ENSBTAG00000038093	*PEG10*	*Y*	*inside the gene (from PEG10 DMR)*
ENSBTAG00000023338	*PEG3*	*Y*	*inside the gene (from PEG3)*
ENSBTAG00000031194	*PHLDA2*	*Y(?)*	*20Kb (from the candidate list)*
ENSBTAG00000026523	*PLAGL1*	*N*	
ENSBTAG00000034645	*PON3*	*N*	
ENSBTAG00000024426	*PPP1R9A*	*N*	

Supplementary Table 4 (Contd). Location of candidate gDMRs with respect to bovine imprinted genes identified by RNA-seq [ref. 9].

ENSBTAG00000015074	*PTGDS*	*Y(?)*	*14Kb (from the candidate list)*
ENSBTAG00000001392	*RDH16*	*N*	
ENSBTAG00000046585	*RTL1*	*Y*	*inside the gene (from the candidate list)*
ENSBTAG00000019437	*SLFN11*	*N*	
ENSBTAG00000021282	*SGCE*	*Y*	*in the promoter (from PEG10 DMR)*
ENSBTAG00000005386	*SLC2A2*	*N*	
ENSBTAG00000008251	*SNRPN*	*Y*	*in the promoter (from SNRPN DMR)*
788661	*USP29^*		
ENSBTAG00000045731	*ZIM2^*	*N*	
ENSBTAG00000048005	*MGC157368*	*N(?)*	*41 Kb (from iAPEG3 DMR)*
100298176	*LOC100298176*	*Y(?)*	*17 Kb (from APEG3 DMR)*
104974975	*LOC104974975*	*N(?)*	*36 Kb (from APEG3 DMR)*
ENSBTAG00000038326	*LOC508098*	*N(?)*	*61 Kb (from APEG3 DMR)*
100848981	*LOC100848941*	*N*	
101907203	*LOC101907203*	*N*	
101907679	*LOC101907679*	*N*	
100849023	*LOC100849023*	*N*	
101905472	*LOC101905472*	*N(?)*	*56 Kb from the candidate list*
Unannotated*	*XLOC_045114*	*N*	
Unannotated*	*XLOC_012439*	*Y(?)*	*7 Kb the from the candidate list*
Unannotated*	*XLOC_052524*	*N*	
Unannotated*	*XLOC_009410*	*N*	

Supplementary Table 5. Promoter methylation and CpG coverage - cow.

Promoter	CGI at the promoter	Oocyte, % methylation	2-4 cells, % methylation	8-16 cells, % methylation	Morula, % methylation	Blastocyst, % methylation	Genomic CpG	Oocyte CpG	2-4 cell CpG	8-16 cell CpG	Morula CpG	Blastocyst CpG
DNMT1s_promoter	YES	1.503	4.303562	1.8821604	18.07971	1.8218844	85	85	47	68	21	63
DNMT1o_promoter	NO	20.238	30.714287	28.333332	35.96154	26.729439	17	17	8	16	12	11
DNMT3A_promoter	NO	99.444	69.99835	70.92949	78.34235	58.147644	59	59	44	59	27	59
DNMT3B_promoter	NO	96.864	41.261574	49.94152	45.454544	43.093567	38	38	19	37	17	35
DNMT3L_promter	NO	27.40232	47.839508	46.074074	48.333336	27.95139	40	40	16	32	13	25
UHRF1_promoter	YES	1.087	4.721442	3.7028904	3.125	2.0527055	171	171	74	97	74	110
TET1_promoter	NO	85.185	31.597223	15.980392	2.3148148	20.308441	21	21	14	13	13	21
TET2_promoter	NO	63.333	64.28571	66.08	65.530304	40.641026	20	20	11	20	11	13
TET3_promoter	NO	96.809	62.59058	66.084946	76.84332	57.882477	52	52	48	52	34	52
TRIM28_promoter	YES	9.343558	3.115367	2.8846154	23.944885	0.9603181	188	188	93	128	60	161
ZFP57_promoter	YES	11	37.185356	20.270271	19.396551	1.5094926	40	40	20	35	23	26
ZNF445_promoter	NO	94.791664	63.995728	74.4124	77.20178	49.60694	33	33	25	31	19	33
DPPA3_promoter	NO	0	20	17.460318	25.897436	19.561403	14	14	10	10	10	7

Genomic CpG: the total number of CpG dinucleotides presented in the given promoter and obtained from genomic assembly
Oocyte (and other stages) CpG: the number of promoter CpGs covered in a given stage

Supplementary Table 5. Promoter methylation and CpG coverage - pig.

Promoter	CGI at the promoter	Oocyte, % methylation	2-4 cells, % methylation	8-16 cells, % methylation	Morula, % methylation	Blastocyst, % methylation	Genomic CpG	Oocyte CpG	2-4 cell CpG	8-16 cell CpG	Morula CpG	Blastocyst CpG
DNMT1s_promoter	YES	60.996883	49.030304	28.468693	36.11111	21.16423	94	64	68	94	29	80
DNMT1o_promoter	NO	11.538462	26.309525	26.40625	18.918919	0	38	13	17	19	19	11
DNMT3A_promoter	YES	17.86834	18.047432	6.316	5.620609	4.3204365	98	50	40	71	37	65
DNMT3B_promoter	NO	66.14489	45.317726	52.264	43.888885	10.813492	33	24	21	22	10	23
DNMT3L_promter	YES	1.3815061	6.327986	7.358	7.674038	7.0254593	163	93	84	142	96	109
UHRF1_promoter	YES	1.737968	2.1436589	5.189	5.855856	2.8973951	87	52	45	74	25	47
TET1_promoter	NO	38.095238	45.65972	52.623	43.546	23.61111	42	10	14	20	15	16
TET2_promoter	YES	0.29761904	0.29411766	2.467	3.368	2.368	131	64	46	87	41	71
TET3_promoter	NO	98.33333	89.58333	82.226	73.747086	27.920227	39	20	27	39	17	30
TRIM28_promoter	YES	15.1005745	9.0494795	4.468006	8.602151	0.15625	117	69	69	113	69	87
ZFP57_promoter	NO	64.583336	50.193798	43.914143	40.357143	9.042553	41	23	24	43	15	25
ZNF445_promoter	NO	8.685446	10.571428	12.526652	20.869566	3.4946237	69	37	36	37	13	47

Genomic CpG: the total number of CpG dinucleotides presented in the given promoter and obtained from genomic assembly
Oocyte (and other stages) CpG: the number of promoter CpGs covered in a given stage

Supplementary Table 6. Lists of pig and cow pluripotency and zygotic genome activation genes.

PIG-Pluripotency GENES (Fig 7a)

KLF4,KLF2,TCF3,TCF3.1,POU5F,ESRRB,KLF5,SOX2,E2F1,SALL4

PIG- Zygote Genome Activation (Fig 7b)

MTRF1L, HEY2, HSF2, MFAP1, OIP5, JKAMP, TGFBR1, ZNF79, C9orf78, MED19, PDHX, ATG4D, EPOR, LONP1-202, EPB41L4A, FAM53C, NUDT1, KDELR2, EIF2AK1, TRRAP, CPSF4, POP7, ABHD11, ARMC5, CD2BP2, NUDT16L1, FBXL16, ITPRIPL1, CEP68, PAPOLG, MTIF2, EPCAM-202, HADHA, NOL10, ADAM17, MTSS1, ATAD2, MTDH, ESRP1, OSGIN2, JPH1, ATP6V1H, DUSP12, CKS1B-204, CRTC2, BCAS2, TRIM33, DNTTIP2, FNBP1L, TNRC6B, JOSD1, HOXC13, NUP107, FGFR1OP2, ATP6V1E1, APPL2, SCYL2, AMDHD1, WDR59, BBS2, OGFOD1, DMWD, SAE1, AKT1S1, ZNF304, MAD2L2, RNF19B, AKIRIN1, RIOK3, MCOLN2, FUBP1, ZYG11A, EPS15, EBNA1BP2, EXOC2, BAG2, APOBEC2, MED20, TBCC, MORF4L1, BLM, FAM177A1, SRP54, ACIN1, MAX, NUMB, AHSA1, GLRX5, SLBP, KDR, USO1, CUL5, STT3A, NUCKS1, BZW2, GNAI1, TPR, FLVCR1, FIGNL1, DEGS1, TFB2M, KLF5, TPP2, ARHGDIA, JMJD6, AMZ2, HOXB3, ANKRD40, NOS2, ERAL1, CRK, ALKBH5, APPL1, PXK, MBD4, HDAC11, ARMC8, FXR1, CLDND1, MORC3, CKS2, KCTD9, DDX54, PTPN11, COX6A1, RNF10, ARL3-202, ARL5A, WDR33, DYNC1I2-205, BARD1, BCS1L, DNAJC21, PDCD6, DSTN, KCNG1, INSIG1, CHCHD3, TMEM185A.

COW-Pluripotency GENES (Fig 8a)

SOX2, NANOG, TCF3, TEX10, ESRRB, KLF5, E2F1, SALL4, SMAD1, STAT3, TDGF1, ZFP42

COW- Zygote Genome Activation (Fig 8b)

WDR33, CYCT, HNRNPA3, HNRNPA3, HAT1, DYNC1I2, GCA, ARL5A, BARD1, BCS1L, SLC9A1, HMGCL, DUSP12, DEDD, GON4L, FLAD1, FLAD1, CRTC2, PRPF3, BCL9, BCAS2, BCAS2, TRIM33, CEPT1, CEPT1, ARHGAP29, DNTTIP2, NEXN, ZYG11A, EPS15, EBNA1BP2, EBNA1BP2, AKIRIN1, GNL2, MIOS, BZW2, BZW2, GNAI1, DLD, TMEM209, CHCHD3, SLC37A3, MRPS33, EZH2, INSIG1, NUDT4, HOXC13, ATF1, TUBA1C, NUP107, CYP27B1, AMDHD1, ACTR6, SCYL2, APPL2, FGFR1OP2, FGFR1OP2, M6PR, NCAPD2, NCAPD2, NCAPD2, ATP6V1E1, MICAL3, JOSD1, MKL1, MKL1, LARP7, LARP7, H2AFZ, H2AFZ, KDR, CEP135, USO1, HAUS3, SLBP, BRD4, BRD4, ATG4D, EPOR, ECSIT, ECSIT, 37316, LONP1, LONP1, 37681, VDAC1, H2AFY, FAM53C, FAM53C, MATR3, PAIP2, TMCO6, TMCO6, HARS, HARS, HARS, G3BP1, EXTL3, FBXO10, TGFBR1, KCTD9, KCTD9, HNRNPK, ISCA1, AUH, CKS2, RNF20, HEY2, HSF2, GOPC, MTRF1L, FAM120B, EPB41L4A, ANP32A, ACIN1, ACIN1, OIP5, OIP5, JKAMP, JKAMP, MAX, AHSA1, AHSA1, ITPRIPL1, EPCAM, MTIF2, PAPOLG, CEP68, HADHA, HADHA, NOL10, ADAM17, ZNF79, C11H9orf16, C11H9ORF78, C11H9ORF78, C11H9ORF78, ESD, RFXAP, RFC3, KLF5, MYCBP2, TPP2, TMCO3, SEC61A2, DHTKD1, OPTN, RSU1, DSTN, GPCPD1, RAE1, AURKA, AURKA, MAPRE1, EIF6, DPM1, DPM1, KCNG1, EIF2C2, MTSS1, ATAD2, ATP6V1H, RPS20, RPS20, SDCBP, JPH1, MTDH, OSGIN2, E2F5, SNX16, CUL5, FXYD6, FXYD6, RRAS2, ARNTL, DEPDC7, PDHX, PDHX, PDHX, MED19, MED19, NUCKS1, DEGS1, TFB2M, TFB2M, METTL13, MAD2L2, ADIPOR1, TSEN15, PRG4, PRG4, FLVCR1, KCNH1, LRBA, CLGN, ANKRD50, PSMD9, DDX54, DDX54, PTPN11, COX6A1, RNF10, HSCB, HSCB, WDR59, ATMIN, BBS2, OGFOD1, OGFOD1, OGFOD1, CDH1, BCKDHA, BCKDHA, CLPTM1, DMPK, DMWD, SAE1, LIG1, LIG1, AKT1S1, AKT1S1, AKT1S1, ZNF304, NOS2, NOS2, NOS2, NOS2, FOXN1, ERAL1, ERAL1, ERAL1, FLOT2, CRK, NUP88, NUP88, ODF4, ALKBH5, ANKRD40, ANKRD40, LUC7L3, ANKRD40, ANKRD40, PHB, HOXB3, MSL1, GHDC, STAT3, ATP6V0A1, ARHGDIA, JMJD6, JMJD6, GGA3, GGA3, AMZ2, UTP15, UTP15, GPBP1,
