# Supplementary material for: DNA methylation changes during preimplantation development reveal inter-species differences and reprogramming events at imprinted genes
Source: Clin Epigenetics. 2020 May 11;12:64. doi: 10.1186/s13148-020-00857-x (PMC7216732; doi:10.1186/s13148-020-00857-x)
Supplement: Supplementary file 2 — Additional file 2: Supplementary Figure 1. Principal Component Analysis (PCA) of replicate PBAT datasets from porcine and bovine gametes and preimplantation embryos, and merged scBS-seq datasets from bovine oocytes. Supplementary Figure 2. CpG coverage rates of merged PBAT or scBS-seq datasets from porcine and bovine gametes and preimplantation embryos. Supplementary Figure 3. The CpG methylation profiles from gametes to blastocyst in pig and cow; additional screenshots to accompany Figure 2a and b. Supplementary Figure 4. Characteristics of regions gaining DNA methylation between the 2-4 and 8-16 cells stages: enrichment analysis for various genomic features; screenshots of representative regions. Supplementary Figure 5. Detection of DNMT1 transcripts originating from oocyte-specific or somatic promoters: screenshots of RNA-seq alignments and intron-spanning alignments. Supplementary Figure 6. Properties of genomic elements differentially methylated in oocytes and sperm in pig and cow. Supplementary Figure 7. Correlations between expression and promoter methylation for pluripotency and zygotic genome activation genes in gametes and preimplantation porcine embryos. Supplementary Figure 8. Correlations between expression and promoter methylation for pluripotency and zygotic genome activation genes in gametes and preimplantation bovine embryos. [file 13148_2020_857_MOESM2_ESM.pdf]

# Supplementary Figure 1

## Porcine

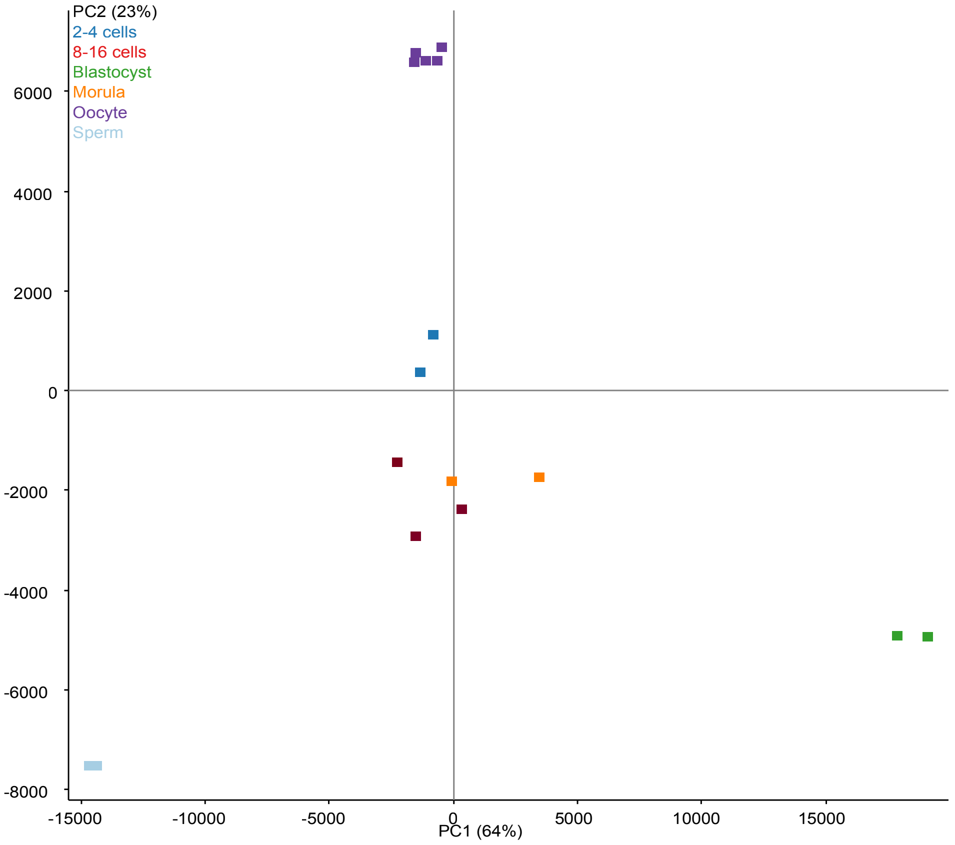


## Bovine

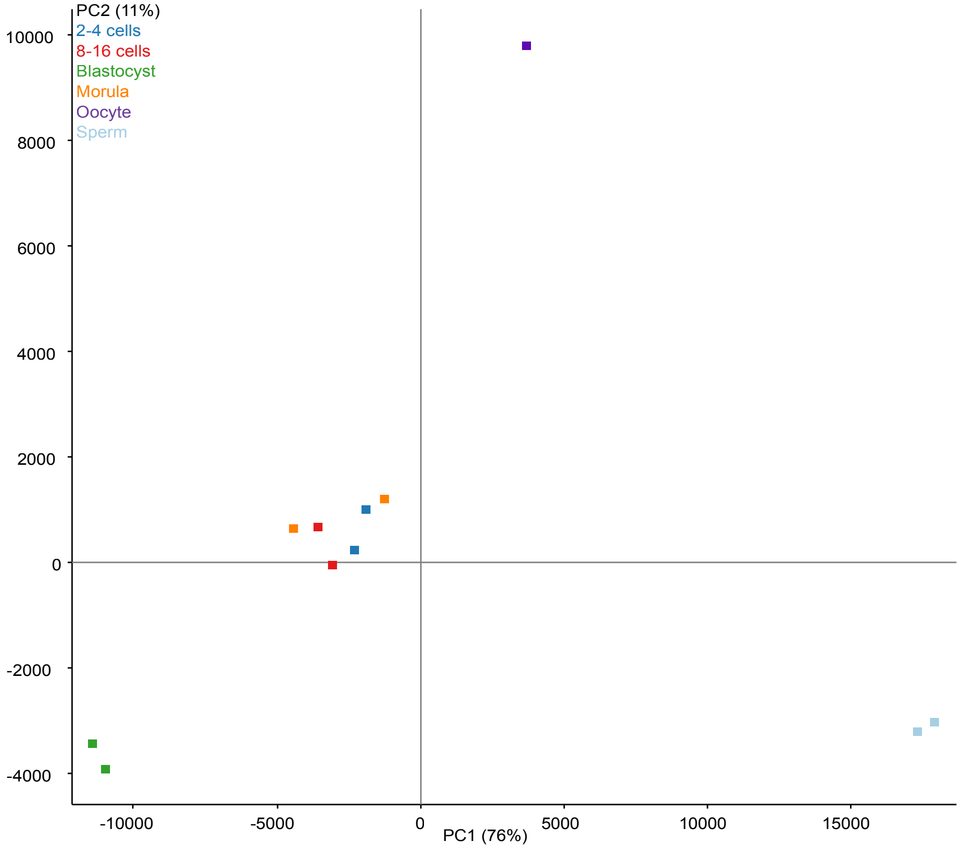

**Supplementary Figure 1.** Principal Component Analysis (PCA) of the data from the replicate PBAT libraries from (upper panel) porcine sperm, oocytes, 2-4 cell embryos, 8-16 cell embryos, morulae and blastocysts; and for bovine (lower panel) sperm, 2-4 cell embryos, 8-16 cell embryos, morulae and blastocysts, together with the merged data for oocytes from 28 scBS-seq datasets. PCA is based on 100-CpG tiles informative in all datasets, with a minimum of 5-CpG observations per tile. The two porcine blastocyst PBAT datasets were previously reported in Canovas, S. et al. 2017 Epigenetic and gene expression changes derived from assisted reproductive technologies can be decreased by reproductive secretions. eLife 6, e23670).

## Supplementary Figure 2

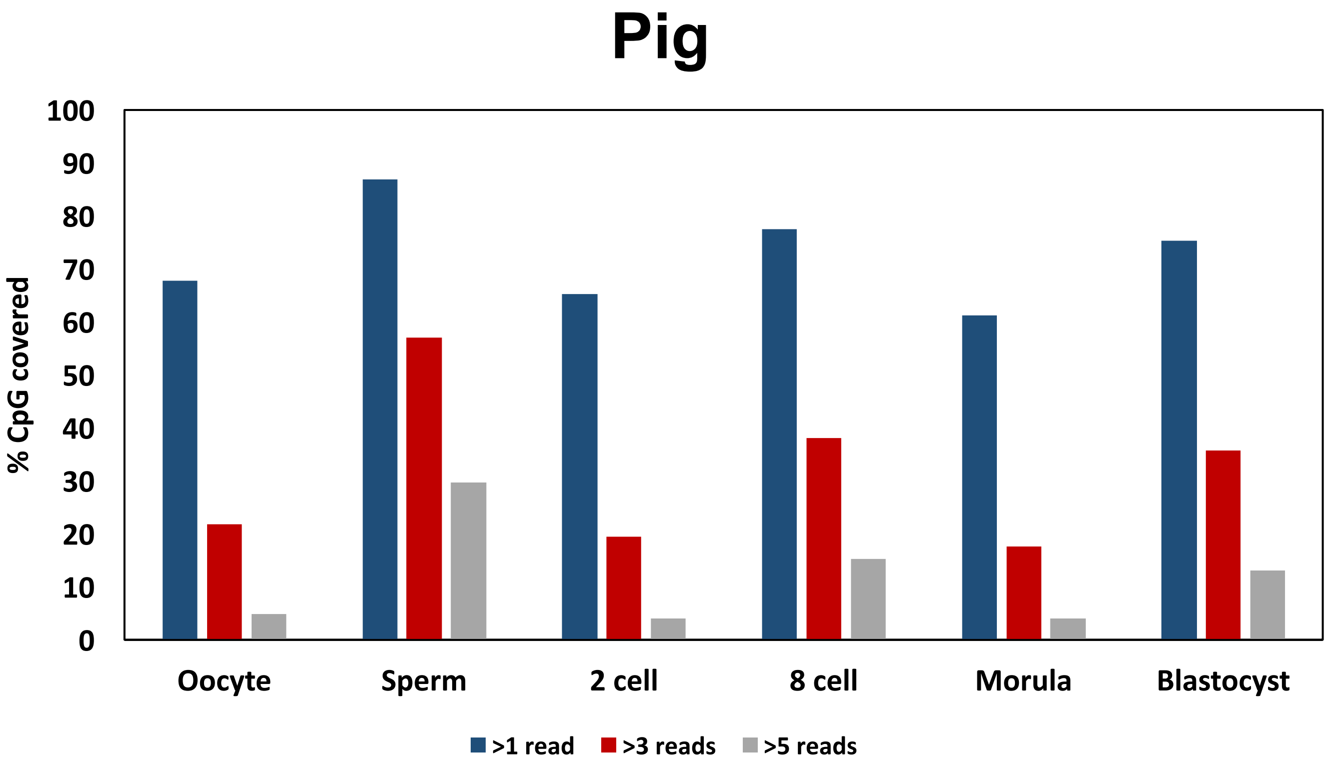


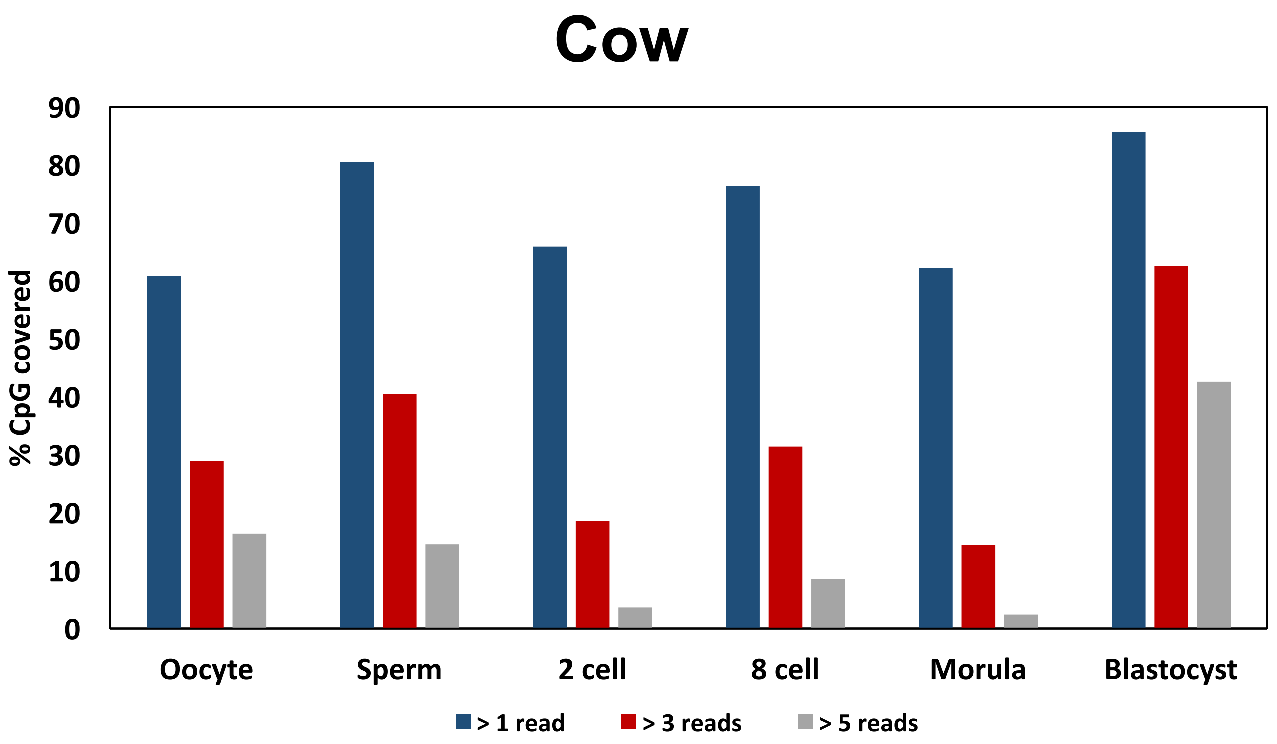

**Supplementary Figure 2.** Percentage of genomic CpG coverage for gametes (oocyte and sperm) and preimplantation embryos in porcine and bovine samples, in each case the data merged from the 2-5 PBAT libraries, or the 28 scBS-seq libraries in the case of the bovine oocytes.

**Supplementary Figure 3.**

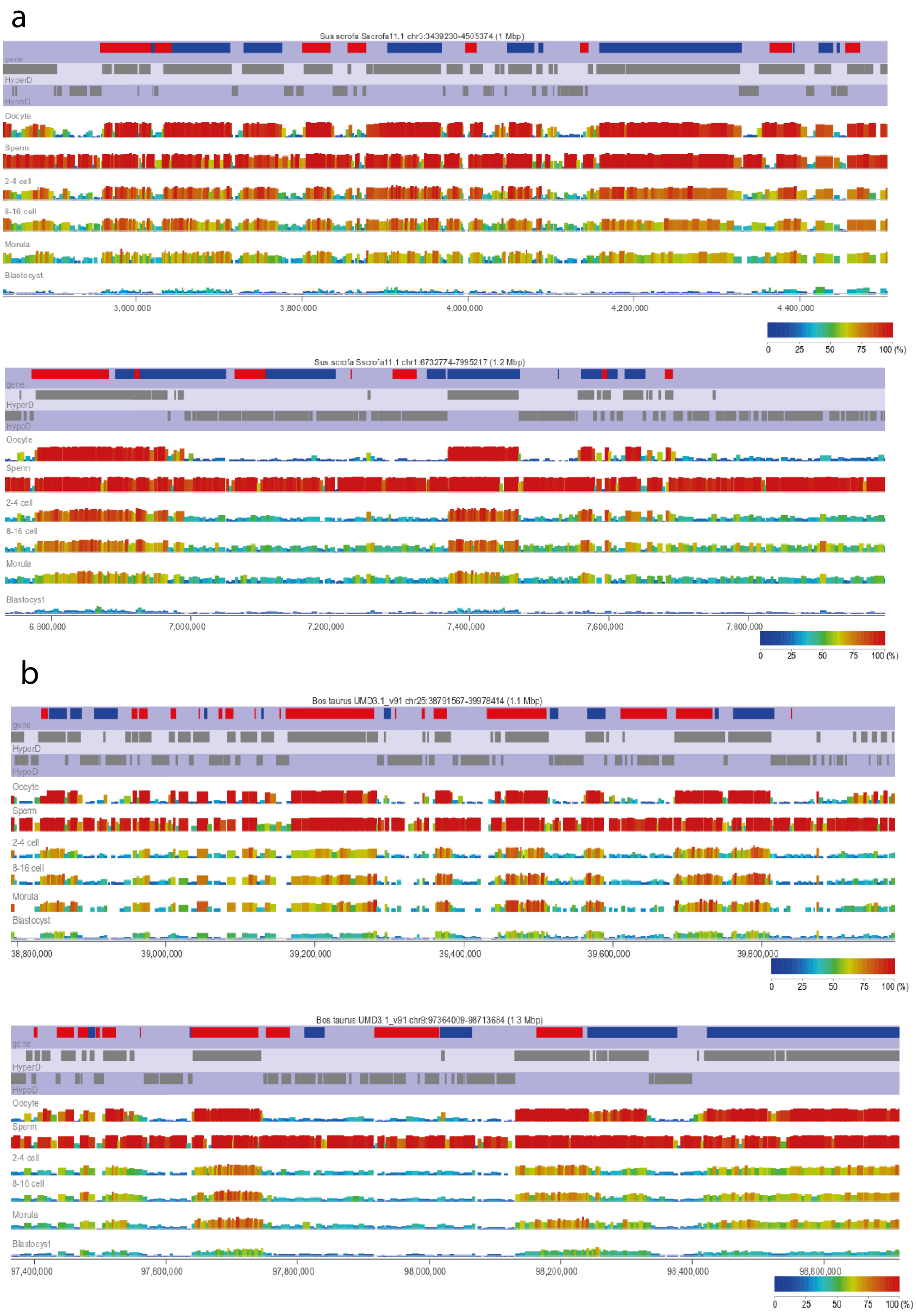

**Supplementary Figure 3.** Changes in the CpG methylation landscape from gametes to blastocyst in pig and cow. Additional screenshots, to accompany Figure 2a and b, from the Seqmonk genome browser at regions of conserved synteny in porcine chromosome 1 and 3 (a) and bovine chromosome 9 and 25 (b) centred on the *ACTIN* and *IGF2R* loci, respectively. For the profiles for each stage, each vertical bar represents the methylation value of a single, non-overlapping 100-CpG tile, with methylation indicated by the height of the bar and the colour-coding according to the indicated scale. At the top of each screenshot, the track 'gene' indicates the location of genes, with those marked red being transcribed from left to right, and those marked blue from right to left; HyperDomains (HyperD) and HypoDomains (HypoD), corresponding to coherent domains with methylation or without methylation, are indicated by red and blue bars, respectively. Each profile represents the merged data of the replicate PBAT libraries per stage, or the merged scBS-seq libraries for the bovine oocytes.

## Supplementary Figure 4.

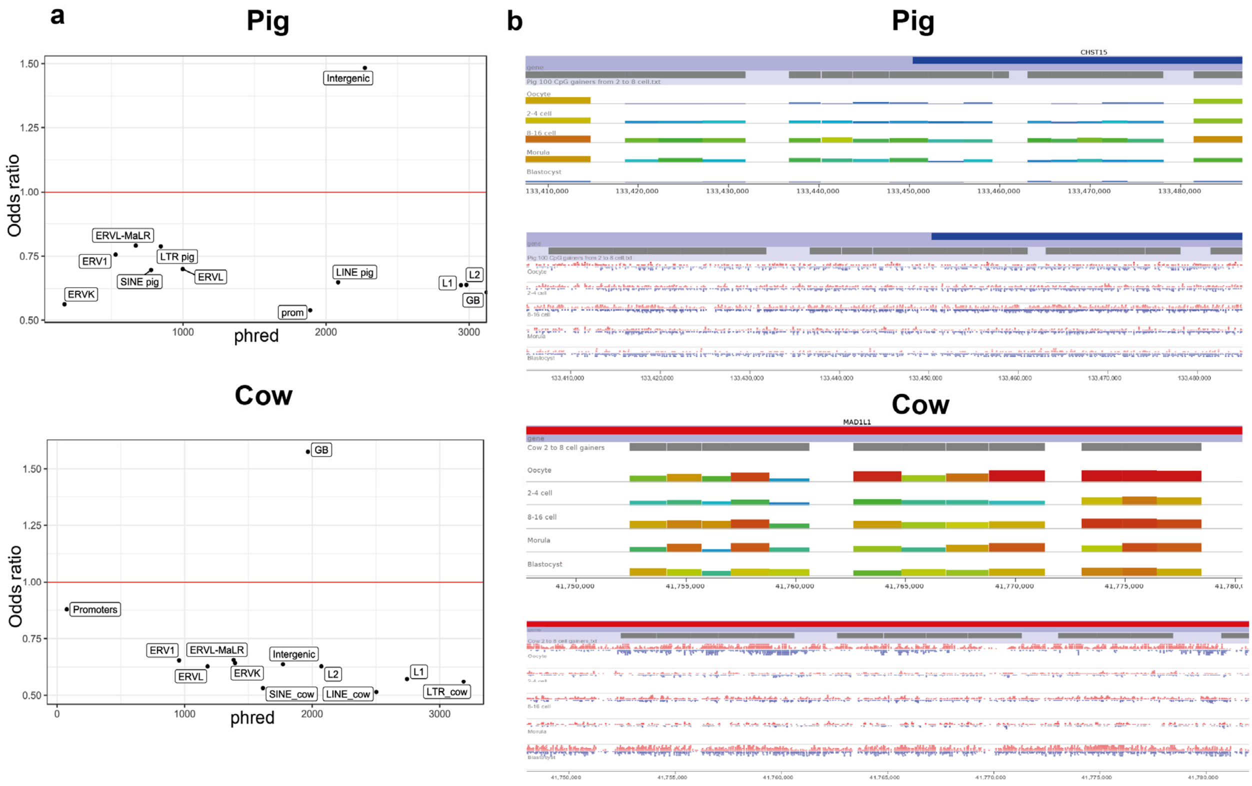


**Supplementary Figure 4.** Characteristics of regions gaining DNA methylation the 2–4 and 8–6 cell stages. a) Enrichment analysis of 100-CpGs tiles in pig (upper) and cow (lower) increasing by > 10% absolute methylation between the 2–4 and 8–6 cell stages. b) Seqmonk genome browser screenshots across pig and cos preimplantation development: upper panels displaying methylation values over 100-CpG tiles (height and colour-coded for % DNA methylation as in Supplementary Figure 3); lower panels the corresponding methylation calls at individual CpG sites, with red dots indicating methylated calls, blue dots unmethylated calls.

## Supplementary Figure 5.

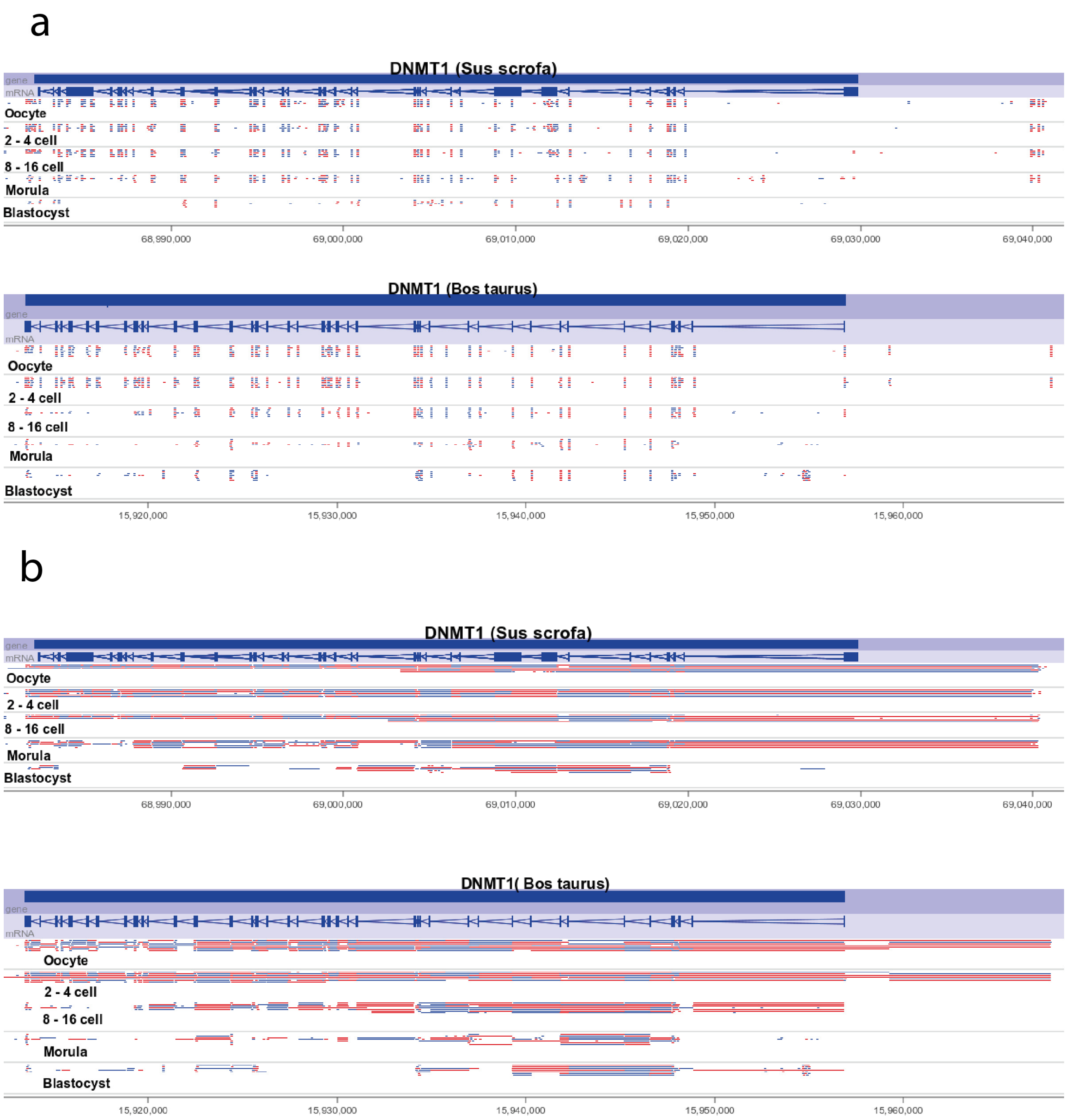


**Supplementary Figure 5.** Detection of DNMT1 transcripts originating from oocyte-specific or somatic promoters in pig and cow oocytes and preimplantation embryos. a) Alignment of the RNA-seq data to exons of the pig (upper) and cow (lower) DNMT1 genes. Note that in both species the genes are transcribed from right to left in the browser. RNA-seq reads are indicated below as red or blue depending on whether they map to the upper or lower strand, but note that the RNA-seq libraries were not made with a stranded method. b) Display of RNA-seq reads that span introns. Note that the annotated transcription start sites in both pig and cow represent the presumed somatic promoter. Reads initiating at an apparent upstream exon and spanning into internal exons detected in oocytes and cleavage-stage embryos in both species represent a putative, unannotated oocyte-specific promoter.

Supplementary Figure 6

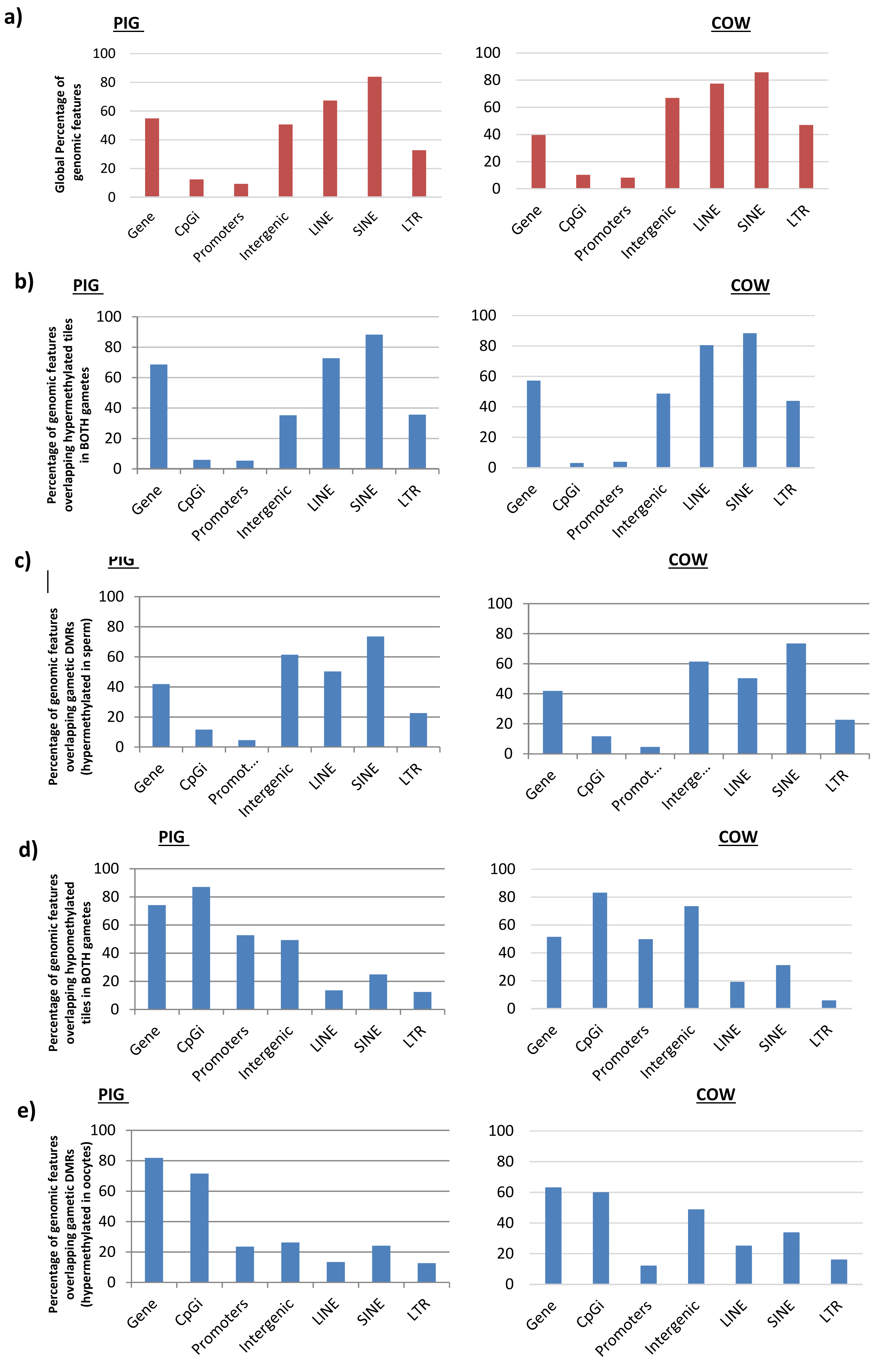

**Supplementary Figure 6.** Properties of genomic features differentially methylated in oocytes and sperm in pig and cow a) percentage enrichment of the indicated genomic features in all 100-CpG tiles; b) tiles hypermethylated (>75%) in both gametes (oocyte and sperm); c) tiles overlapping gametic Differentially Methylated Regions -DMRs- (hypermethylated only in sperm); d) tiles hypormethylated (<25%) in both gametes (oocyte and sperm); e) tiles overlapping gametic Differentially Methylated Regions -DMRs- (hypermethylated only in oocytes).

**Supplementary Figure 7**

a) Correlations in porcine pluripotency genes

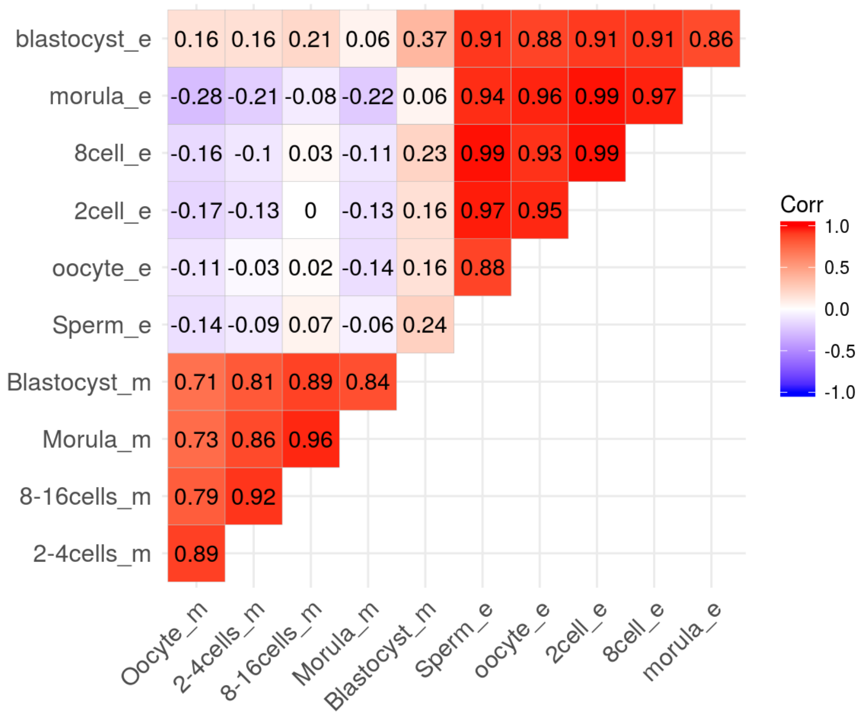

## b) Correlations in zygotic genome activation porcine genes

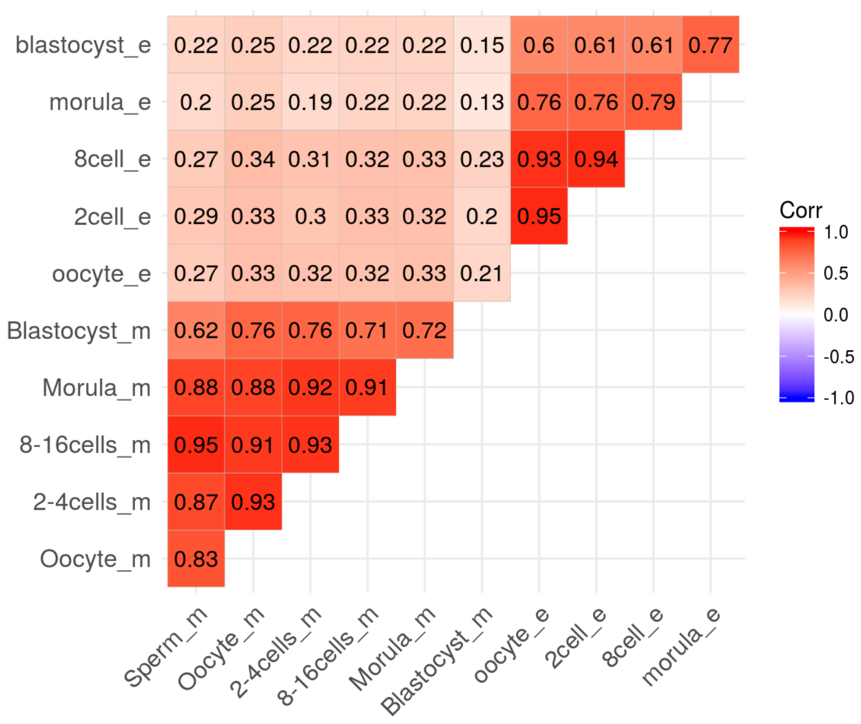


**Supplementary Figure 7.** Correlations between expression and promoter methylation for pluripotency (a) and zygotic genome activation (b) genes in gametes and preimplantation porcine embryos (e: expression; m: methylation; corr: correlation).

**Supplementary Figure 8**

a) Correlations in cow pluripotency genes

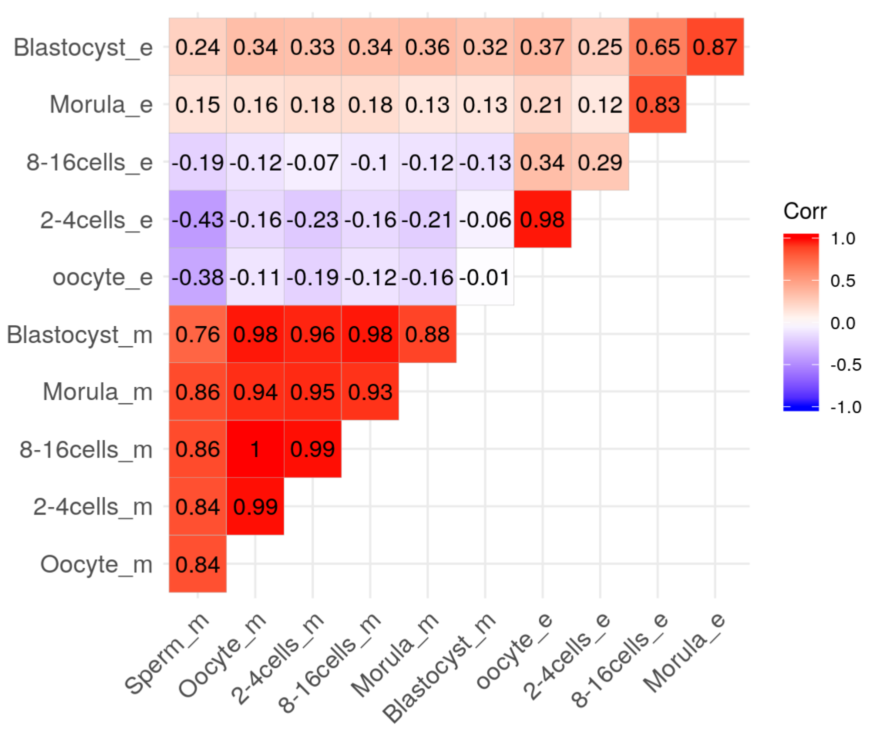

b) Correlations in cow zygote genome activation genes

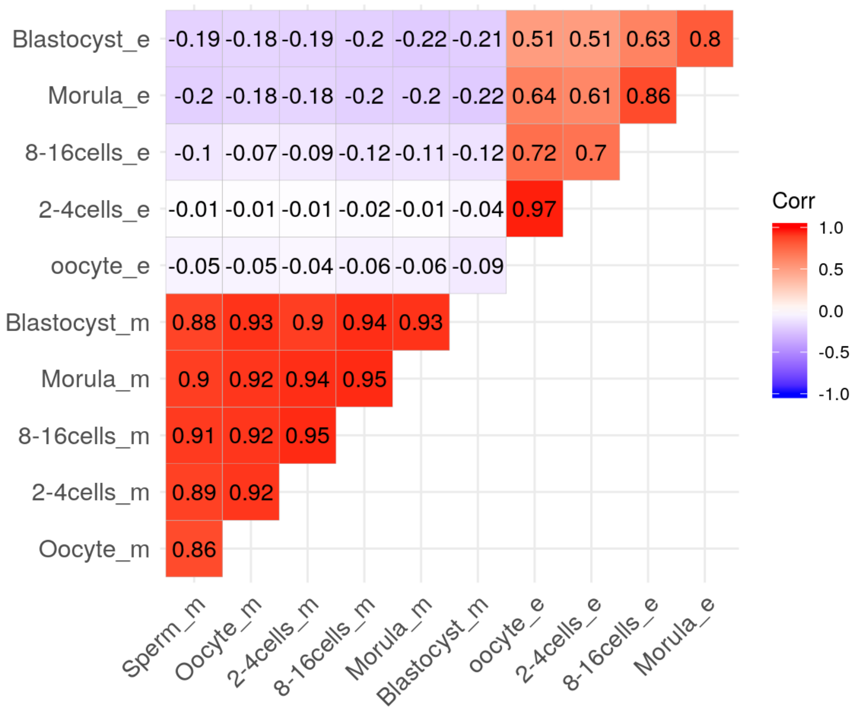


**Supplementary Figure 8.** Correlations between expression and promoter methylation for pluripotency (a) and zygotic genome activation (b) genes in gametes and preimplantation bovine embryos (e: expression; m: methylation; corr: correlation).
